# Blood transfusion in the care of patients with visceral leishmaniasis: a review of practices in therapeutic efficacy studies

**DOI:** 10.1093/trstmh/trae018

**Published:** 2024-05-01

**Authors:** Prabin Dahal, Sauman Singh-Phulgenda, James Wilson, Glaucia Cota, Koert Ritmeijer, Ahmed Musa, Fabiana Alves, Kasia Stepniewska, Philippe J Guerin

**Affiliations:** Infectious Diseases Data Observatory (IDDO), OX3 7LG, Oxford, UK; Centre for Tropical Medicine and Global Health, Nuffield Department of Medicine, University of Oxford, OX3 7LG, Oxford, UK; Infectious Diseases Data Observatory (IDDO), OX3 7LG, Oxford, UK; Centre for Tropical Medicine and Global Health, Nuffield Department of Medicine, University of Oxford, OX3 7LG, Oxford, UK; Infectious Diseases Data Observatory (IDDO), OX3 7LG, Oxford, UK; Centre for Tropical Medicine and Global Health, Nuffield Department of Medicine, University of Oxford, OX3 7LG, Oxford, UK; Instituto René Rachou (IRR), Fiocruz Minas, Augusto de Lima Avenue, 1517, Barro Preto, Belo Horizonte, Minas Gerais Brazil, ZIP code 30.190-002, Brazil; Médecins Sans Frontiéres, Plantage Middenlaan 14, 1018 DD Amsterdam, Netherlands; Institute of Endemic Diseases, University of Khartoum, Khartoum 11111, Sudan; Drugs for Neglected Diseases initiative, CH1202, Geneva, Switzerland; Infectious Diseases Data Observatory (IDDO), OX3 7LG, Oxford, UK; Centre for Tropical Medicine and Global Health, Nuffield Department of Medicine, University of Oxford, OX3 7LG, Oxford, UK; Infectious Diseases Data Observatory (IDDO), OX3 7LG, Oxford, UK; Centre for Tropical Medicine and Global Health, Nuffield Department of Medicine, University of Oxford, OX3 7LG, Oxford, UK

**Keywords:** anaemia, blood, haemoglobin, Kala-Azar, systematic review, transfusion

## Abstract

Blood transfusion remains an important aspect of patient management in visceral leishmaniasis (VL). However, transfusion triggers considered are poorly understood. This review summarises the transfusion practices adopted in VL efficacy studies using the Infectious Diseases Data Observatory VL clinical trials library. Of the 160 studies (1980–2021) indexed in the IDDO VL library, description of blood transfusion was presented in 16 (10.0%) (n=3459 patients) studies. Transfusion was initiated solely based on haemoglobin (Hb) measurement in nine studies, combining Hb measurement with an additional condition (epistaxis/poor health/clinical instability) in three studies and the criteria was not mentioned in four studies. The Hb threshold range for triggering transfusion was 3–8 g/dL. The number of patients receiving transfusion was explicitly reported in 10 studies (2421 patients enrolled, 217 underwent transfusion). The median proportion of patients who received transfusion in a study was 8.0% (Interquartile range: 4.7% to 47.2%; range: 0–100%; n=10 studies). Of the 217 patients requiring transfusion, 58 occurred before VL treatment initiation, 46 during the treatment/follow-up phase and the time was not mentioned in 113. This review describes the variation in clinical practice and is an important initial step in policy/guideline development, where both the patient's Hb concentration and clinical status must be considered.

## Introduction

Visceral leishmaniasis (VL) is the most severe of the three forms of leishmaniasis and is fatal without treatment. The disease typically presents insidiously and is characterised by protracted fever, hepatosplenomegaly and weight loss, often with evolving anaemia, leukopaenia and thrombocytopaenia leading to pancytopaenia.^1^ At presentation, moderate anaemia around 7–10 g/dL is common but can evolve into severe anaemia.^1^ While the underlying mechanism for the onset of anaemia in VL is likely multifactorial, the literature suggests the main cause is due to macrophage-induced haemolysis in the spleen (‘splenic sequestration’, ‘splenic haemophagocytosis’).^2,3^

In patients presenting with VL, especially when accompanied by signs of anaemia (typically shortness of breath, fatigue, weakness, light-headedness, occasionally chest pain), transfusion of blood products may be clinically indicated. The 2010 WHO technical report on leishmaniasis suggests undertaking transfusion in patients with severe anaemia (defined as haemoglobin≤5g/dL).^4^ Red blood cell (RBC) transfusion may also be indicated in cases of acute blood loss due to invasive procedures such as splenic aspiration or venous catheterisation, or due to spontaneous haemorrhage from VL-related bleeding diathesis. Bleeding risk is exacerbated in VL patients as the disease can lead to alterations of hepatic coagulation factors and thrombocytopaenia.^5^ Therefore, operational manuals from national control programmes suggest that haematological factors are taken into consideration prior to splenic aspirations (or completely contra-indicate splenic aspiration) and that blood transfusion services are in place for the management of acute blood loss.^5–9^

Despite the importance of transfusion in the management of VL patients, the actual practice in clinical trials is poorly reported and remains largely unclear. In this review, we aim to collate information regarding practices adopted to initiate blood transfusion in therapeutic efficacy studies of VL using the open-source Infectious Diseases Data Observatory (IDDO) VL clinical trials library.^10^

## Materials and Methods

### Information sources and search strategy for clinical trials

This review synthesises data from all published studies currently indexed in the IDDO VL clinical trials library (referred as the IDDO VL library henceforth) of prospective therapeutic studies from 1980 until November 2021.^10^ The IDDO VL library is updated on a periodic basis; the details of the search strategy adopted are described elsewhere.^11^ Data on the following aspects of the included studies were extracted: the number of patients enrolled, the number of patients who received a transfusion (any blood products), details of the timing of transfusion (prior to treatment initiation, during the treatment phase or during the follow-up phase after discharge), the details of the blood products transfused (whole blood, packed RBCs, plasma or platelets), the haematological measurements adopted as a part of the study inclusion/exclusion criteria and details regarding any parameters used as a transfusion trigger, including the haemoglobin/haematocrit threshold adopted.

### Data summary and analysis

A descriptive summary of the data extracted is presented. The median proportion of patients reported to have received a blood product is presented along with the range and interquartile range (IQR). No formal meta-analysis was undertaken because of the large heterogeneity in the transfusion practices adopted. All statistical analyses and graphical presentations used R software.^12^

### Risk of bias assessment

The risk of bias assessment in studies included in this review was carried out using the Cochrane Risk of Bias tool for randomised controlled trials.^13^ Risk of bias in non-randomised studies was carried out using the ROBINS-I tool.^14^ Two reviewers (PD and SSP) independently assessed the risk of bias.

## Results

The IDDO library has currently indexed 160 publications (35 763 patients; 1980–2021). There were 108 (67.5%) studies from the Indian subcontinent, 27 (16.9%) from East Africa, nine (5.6%) from the Mediterranean region, seven (4.4%) from South America, five (3.1%) from Central Asia (the Middle East) and four (2.5%) were multiregional studies. The haematological measures adopted for patient inclusion are presented in Table 1. The minimum haemoglobin concentration required for inclusion was 3 g/dL in eight (5.0%) studies, >3–5 g/dL in 38 (23.8%) studies, >5–7 g/dL in 22 (13.8%) studies and it was not mentioned in the remaining 92 (57.5%) studies (including one study that described severe anaemia as an exclusion criterion). The range of further haematological parameters considered at inclusion are presented in Table 1 and Supplemental Table S1.

**Table 1.** Haematological parameters considered in defining inclusion/exclusion criteria for patient enrolment (n=160 studies)

Haematological parameters	Number of studies (number of patients)	% (n=160 studies)
Minimum haemoglobin concentration		
3 g/dL	8 (n=1804)	5.0%
3 to ≤5 g/dL	38 (n=11 452)	23.8%
5 to ≤7 g/dL	22 (n=2644)	13.8%
Not mentioned^a^	92 (n=19 863)	57.5%
Prothrombin time (above control values)		
4 s	9 (n=1277)	5.6%
5 s	14 (n=5599)	8.8%
15 s	2 (n=631)	1.3%
20 s	1 (n=89)	0.6%
Prothrombin activity<40%	1 (n=57)	0.6%
International normalisation ratio (INR)>2	1 (n=378)	0.6%
Not mentioned	132 (27 732)	82.5%
Minimum platelets concentration		
4000/μL	1 (n=30)	0.6%
5000/μL	1 (n=60)	0.6%
20 000/μL	1 (n=378)	0.6%
30 000/μL	2 (n=38)	1.3%
40 000/μL	22 (n=7432)	13.8%
50 000/μL	19 (n=2681)	11.9%
60 000/μL	1 (n=230)	0.6%
75 000/μL	1 (n=120)	0.6%
80 000/μL	4 (n=50)	2.5%
Not mentioned	108 (n=24 274)	67.5%
Minimum WBC count		
<750/μL	1 (n=412)	0.6%
<1000/μL	18 (n=7086)	11.3%
<2000/μL	8 (n=756)	5.0%
Not mentioned^b^	133 (n=27 513)	83.1%

a Of the 92 studies that did not mention the haemoglobin threshold, one stated anaemia as exclusion criteria without stating the threshold.

b Further 19 studies that reported granulocytes only and 2 studies with unclear units of measurements are grouped as Not mentioned.

Abbreviation: WBC, white blood cell.

### Characteristics of the studies describing blood transfusion

Overall, of the 160 studies, 7/108 studies from the Indian subcontinent, 6/27 studies from East Africa, 2/7 studies from South America, 1/5 studies from Central Asia, none of the nine studies from the Mediterranean region and none of the four multiregional studies reported any information on blood transfusion. The description of blood transfusion was explicitly reported in only 16 (10.0%, 16/160) studies (n=3459 patients; 1984–2018) (Table 2). When considered by time period, eight (11.3%) of the 71 studies published during 1980–1999, five (10.4%) of the 48 studies published during 2000–2009 and three (7.3%) of the 41 studies published during 2010–2021 presented a description of blood transfusion.

**Table 2.** Description of transfusion in clinical studies of visceral leishmaniasis (n=16 studies)

Author-year	Country	Drug regimen	Total enrolled	Number of transfusions	Transfusion triggers	Transfused product	Description of transfusions as reported in the original publication	Number of patients requiring transfusion at baseline	Transfusion during treatment/follow-up phase
Rees-1984^22^	Kenya	SSG	16	1	Unclear if there were any specific rules (the transfused patient had Hb of 3.2 g/dL)	Blood	In one pregnant patient with a haemoglobin of 3.2 g/dl, 2 pints of blood were given concomitantly with the commencement of SSG	–	Reports transfusion during treatment; number of patients not mentioned
Thakur-1984^15^	India	SSG	750	4	4 g/dL + poor health OR haemorrhage	Blood	If haemoglobin was <4.0 g/dL and the general condition of the patient was poor, a blood transfusion was given. Blood transfusions were given to all cases with haemorrhage. Four cases of *cancrum oris* were encountered in the early phase of the epidemic when drugs were scarce. With specific treatment, general management with blood transfusion and oral protein supplements and crystalline penicillin, *cancrum oris* improved	–	–
Thakur-1988^44^	India	SSG	371	0	Not mentioned	Transfusion not required	Patients were selected so that they did not have a haemoglobin concentration of <3 g/dL, no blood transfusion was required for any patient	0	0
Thakur-1991^45^	India	Pentamidine; Pentamidine + SSG	312	Not mentioned	Hb<3 g/dL	Blood	Patients whose haemoglobin level was <3 g/dL were given a blood transfusion, and when the haemoglobin level improved beyond 3 g/dL, they were included in the trial	Transfused at baseline; number of patients not mentioned	–
Dietze-1993^46^	Brazil	ABCD	20	Not mentioned	Not mentioned	Red cells	Transfusions of red blood cells during therapy was described	–	Transfused during treatment; number of patients not mentioned
Thakur-1993^24^	India	Amphotericin B	50	4	Hb<4 g/dL + pre-existing haemorrhagic problems	Blood	Blood transfusions were given if haemoglobin was <4 g/dL, or if a patient had any haemorrhagic problems like epistaxis. Epistaxis occurred in 4 patients and it was treated with blood transfusions (Table 1 of the publication indicates transfusions occurred before treatment initiation as no epistaxis were reported during the study)	4	0
Berhe-1999^26^	Ethiopia	PA	23	Not mentioned	Severe anaemia	Blood	Blood transfusion was given to patients with severe anaemia and the post-transfusion haemoglobin value among the two groups was not different	Not mentioned	Not mentioned
Thakur-1999^19^	India	AMBd	938	56	Hb<5 g/dL	Blood	Patients whose haemoglobin level was <5 g/dL were given blood transfusions first and only when the haemoglobin reached 5 g/dL was treatment for visceral leishmaniasis started. Blood transfusion was required in 54 (6%) patients before the start of treatment. Anaemia improved with treatment but in 2 patients, haemoglobin dropped after treatment and they required blood transfusions	54	2
Moore-2001^20^	Kenya	SSG	102	43	Anaemia	Blood	Blood transfusions were available for anaemic patients. Patients required transfusions during and after treatment; described in Table 2 of the manuscript^20^	Not mentioned	43
Haidar-2001^25^	Yemen	SSG	32	24	Not mentioned	Blood	Blood transfusions were required for 24 patients (73%)	Not mentioned	Not mentioned
Mueller-2008^47^	Uganda	AMBd; PA	371	Not mentioned	Severe anaemia	Blood	Blood transfusions for severe anaemia were given, if necessary	Not mentioned	Not mentioned
Das-2009^48^	India	AMBd; Pentamidine	82	Not mentioned	Hb<6 g/dL	Blood	In cases of VL who had severe anaemia (Hb<6 g/dL), before excluding such patients from the study, an effort was undertaken to increase their haemoglobin level by giving fresh blood transfusions as required	Transfused at baseline; number of patients not mentioned	Not mentioned
Adam-2009^23^	Sudan	SSG	42	42	Severe anaemia	Blood	All 42 patients received blood transfusions for severe anaemia	Not mentioned	Not mentioned
Thakur-2010^18^	India	Amphotericin B	230	Not mentioned	Hb<5 g/dL	Whole blood/platelets	If patients had haemoglobin levels <5 g/dL or a thrombocyte count<65 000 cells/μL, whole blood transfusions or platelet transfusions were given, respectively. If these two parameters reached acceptable levels, only then were the patients included in the trial	Transfused at baseline; number of patients not mentioned	Not mentioned
Cota-2014^17^ (HIV negative)	Brazil	PA; AMBd; L-AmB	46	19	Measured Hb/platelets/INR + clinical instability/bleeding	Red cells or plasma or platelets	In this study, all types of blood products were accounted for between baseline and the end of hospitalisation for VL treatment. Specific transfusion rules: platelets: <20 000 + active bleeding or <50 000 before performing invasive procedures; red cells: severe anaemia or Hb<8 g/dL + clinical instability; plasma: INR>1.5 + active bleeding (personal communication: Dr Glaucia Cota)	Not mentioned	Not mentioned
Cota-2014^17^ (HIV positive)	Brazil	PA; AMBd; L-AmB	44	23	Measured Hb/platelets/INR + clinical instability/bleeding	Red cells or plasma or platelets	In this study, all types of blood products were accounted for between baseline and the end of hospitalisation for VL treatment. Specific transfusion rules: platelets: <20 000 + active bleeding or <50 000 before performing invasive procedures; red cells: severe anaemia or Hb<8 g/dL + clinical instability; plasma: INR>1.5 + active bleeding (personal communication: Dr Glaucia Cota)	Not mentioned	Not mentioned
Mbui-2018 ^21^	Kenya, Uganda	Miltefosine	30	1	Not mentioned	Not mentioned	A case of ‘transfusion reaction’ was reported. This was considered an important medical event by the investigator, occurring on day 203 after the start of treatment	Not mentioned	1

Abbreviation: ABCD, amphotericin b colloidal dispersion; AMBd, amphotericin b deoxycholate; Hb, haemoglobin; INR, international normalisation ratio; L-AmB, liposomal amphotericin b; PA, pentavalent antimony; SSG, sodium stibogluconate.

Of the 16 studies that explicitly reported the occurrence (or the absence) of transfusion, VL patients with HIV coinfection were included in two studies (n=113 patients), were excluded in four studies (n=384 patients) and their inclusion/exclusion was unclear in the remaining 10 studies (n=2962 patients). Pregnant women were included in three studies (n=996 patients), excluded in four studies (n=304 patients) and their inclusion/exclusion was unclear in the remaining nine studies (n=2159 patients).

### Transfused products and transfusion triggers (n=16 studies)

Transfusion was not required for any patient in one study and transfusion of either whole blood or other blood products was reported in the remaining 15 studies (Table 2). Of the latter 15 studies, transfusion of blood (without specifying if whole blood or the blood components were transfused) was reported in 12 studies, one study transfused RBCs and two studies reported transfusing either blood (without making a distinction if whole blood or packed RBCs) or platelets.

Transfusion was initiated solely based on the measured concentration of haemoglobin (or anaemia status) or platelet concentrations in nine studies (Table 2). In two studies from India, the transfusion trigger was a combination of measured haemoglobin concentration and existing clinical condition such as epistaxis or poor health.^15,16^ In a study from Brazil,^17^ the measured concentration of red cells or international normalisation ratio (INR) in addition to the clinical condition of the patient was adopted; transfusion of RBCs was undertaken if a patient had severe anaemia (haemoglobin<8 g/dL) along with clinical instability, and plasma transfusion was undertaken if the INR>1.5 and the patient had active bleeding. The criteria used for blood transfusion was not stated in the remaining four studies (Table 2).

The haemoglobin threshold used as a transfusion trigger was 3 g/dL in two studies, 4 g/dL in two studies, 5 g/dL in two studies, 6 g/dL in one study and 8 g/dL in one study. Anaemia or severe anaemia status was used without reporting the haemoglobin threshold in four studies and the threshold was not described in four studies (Table 2).

Of the two studies that reported data on platelet transfusion, a threshold of <65 000 cells/μL was used in a study from India,^18^ and in another study from Brazil^17^ a threshold of <50 000 cells/μL was adopted among those who were given prophylactic transfusion before undertaking invasive procedures and a threshold of <20 000 cells/μL for patients with active bleeding (Table 2).

### The number of blood transfusions reported (n=10 studies)

**Figure 1.**
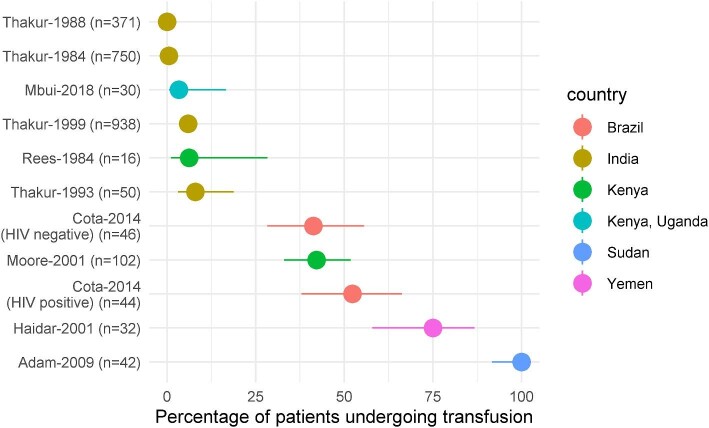
Proportion of patients undergoing transfusion in 10 studies that clearly reported the transfusion status. Cota-2014 is presented separately by the HIV status of the patients.

The number of patients who received a transfusion was clearly reported in only 10 studies (2421 patients enrolled); a total of 217 patients received blood transfusions (the total number of transfusion episodes could not be discerned). Of these 217 patients, 58 transfusions occurred before initiation of antileishmanial therapies,^16,19^ 46 patients underwent transfusion during the treatment or the follow-up phase^19–21^ and the time when transfusion occurred was not reported for the remaining 113 patients (Table 2). Overall, from the 10 studies that clearly reported the number of patients who required a transfusion, the median proportion of patients who received a transfusion at any time point in the study was 8.0% (IQR: 4.7% to 47.2%; range: 0–100%; Figure 1).

### Transfusion by patient risk-group

**Pregnancy**: Transfusion among pregnant women was described in two studies. In a study from Kenya, one patient (1/16) required transfusion and the patient had a haemoglobin concentration of 3.2 g/dL.^22^ In another study in pregnant women from Sudan, all patients (100%, 42/42) were transfused based on severe anaemia status.^23^

**Children:** In three studies that explicitly enrolled children aged <15 y (112 enrolled), 29 patients required transfusion (Table 2 and Supplemental Tables S1 and S2). The criteria for transfusion was haemoglobin concentration <4 g/dL along with a pre-existing haemorrhagic problem in a study from India.^24^ This was not mentioned in a study from Yemen^25^ and in a multicentre study conducted in Kenya and Uganda.^21^

**VL-HIV coinfection:** VL patients with HIV coinfection were enrolled in two studies. In a study from Brazil, 23/44 (52.2%) patients required transfusion.^17^ In another study from Ethiopia, the number of patients requiring transfusion was not reported (23 patients enrolled; the number requiring transfusions was not reported).^26^

### Risk of bias assessment in the studies included (n=16 studies)

Of the 16 studies that provided descriptions of blood transfusion, four were randomised and 12 were non-randomised. The four randomised studies were judged to be at high/unclear risk of bias for the blinding domain, low or unclear risk of bias for the sequence generation and allocation concealment domains. Of the 12 non-randomised studies, all of them were either open label or the description was unclear, bias in participants selection was considered low/moderate in 10 studies, high in one study and unclear in one study. Further details regarding the bias assessment are presented in Supplemental Tables S3 and S4.

## Discussion

From all published studies indexed in the IDDO VL library (1980–2021), reporting of information regarding blood transfusion was not explicit in the majority of studies. This could be partly due to the exclusion of patients with severe anaemia or patients with advanced disease status in standard VL efficacy studies (Table 1). For example, 46 of the 160 therapeutic efficacy studies excluded patients with haemoglobin concentration ≤5 g/dL. Such criteria adopted will lead to the exclusion of severe patients and patients with severe anaemia who may be more likely to require transfusion. From the 16 studies that clearly reported occurrences (or absences) of blood transfusion, the criteria adopted varied between the studies. Most of the studies reported initiating blood transfusion based on measured haemoglobin concentration or anaemia status; the haemoglobin concentration used as a transfusion trigger ranged from 3 to 8 g/dL. This wide variation is particularly relevant as studies in the non-VL context have pointed towards the lack of benefit of blood transfusion in preventing mortality when transfusion is initiated at haemoglobin concentration >4 g/dL among the paediatric population.^27,28^

In addition to the assessment of haemoglobin concentration, it is also important to consider further haemodynamic stability and the clinical history of patients, such as heart conditions, when considering the adoption of a transfusion threshold.^29^ However, only three studies included in this review reported carrying out transfusion using a combination of measured haemoglobin concentration and further criteria such as clinical stability, poor health or active bleeding such as epistaxis (Table 2). This is particularly relevant as the ability to tolerate anaemia can partly depend on the speed of its evolution, as compensatory mechanisms can enable relatively severe degrees of anaemia to be tolerated if it develops over a prolonged duration.^30^ VL primarily affects the poor and marginalised populations with limited access to healthcare, leading to a prolonged duration of illness prior to presentation. For example, the mean duration of illness prior to clinical presentation in studies included in the IDDO library was >100 d in the studies published during the 1980s and approximately around 50 d in the studies published during the 2010s.^31^ As the disease itself often evolves insidiously over weeks or months, and patients often receive care late in the disease course, anaemia evolves over a prolonged period.^32^ Therefore, in VL patients, it can be anticipated that compensatory mechanisms will have led to a physiological adaptation to anaemia. However, acute anaemia arising as a result of acute bleeding occurring due to complications during splenic puncture or postpartum haemorrhage among pregnant VL patients^33^ can overwhelm the compensatory mechanisms of the body and can be fatal, thus requiring immediate transfusion.

Caution is urged when transfusing patients who present with severe acute malnutrition (a common feature of VL patients), as fluid overload and respiratory impairment are a recognised and feared complication in patients with hypoalbuminaemia.^34^ Further caution is required when undertaking transfusion as assessment of transfusion-related safety risks remains crucial. In the studies included in this review, one case of transfusion reaction was reported,^21^ but specific details on the nature of this adverse event were not presented. In other studies included in this review, no reports on the occurrence of transfusion reactions or transfusion-associated risks were presented. In general, transfusion reactions are estimated to occur up to 1 per 100 transfusions^35^ and the risks associated with transfusion in the context of VL are currently not well understood.

From a relatively limited set of studies that reported details on transfusion, the median proportion of patients who received a transfusion was 8% (n=10 studies) in clinical trials settings. Patients with severe anaemia or those with severe disease and pre-existing comorbidities are excluded in standard therapeutic efficacy studies, leading to the inclusion of patients with mostly uncomplicated VL (Table 1).^36^ Therefore, the expected incidence of transfusion in routine clinical practice is likely to be higher than that observed in clinical trials, especially among those patients presenting with advanced disease. This suggests there might be a substantial economic/logistical cost to the healthcare facilities arising from the requirement of transfusion in the management of VL patients. The cost could also be further increased as a central cause of anaemia may require multiple transfusions, leading to increased expenditure and the management of potential safety risks associated with transfusion alone.^37–39^

This review has several limitations. Of the 160 clinical studies in the IDDO library, the majority of the studies did not present any information related to the occurrence of transfusion or the provision of it. And when reported, data on the actual number of patients requiring transfusion or the total number of episodes of transfusion were also not clear. This may be due to the fact that blood transfusion is not the primary focus in the majority of the efficacy studies. Therefore, lack of details regarding transfusion and lack of disaggregation of outcomes by the transfusion status prevented a thorough assessment of the impact of transfusion on therapeutic outcomes. Another important aspect that can potentially influence the impact of transfusion on treatment outcome is the condition of the health services where the patients were treated, including the possibility of different local guidelines for transfusions. For example, three studies reported undertaking haematological profile correction prior to patient enrolment without providing further details.^18,40,41^ The impact of such a strategy (i.e. the influence of timing of haematological profile correction on treatment outcomes) currently remains unclear. Finally, from the studies included in this review, it was also not possible to assess if some patient groups were more or less likely to require transfusion than others. These important aspects of VL patient management warrant further investigation.

A checklist of items for reporting data related to transfusion is proposed in Box 1. Adoption of such a checklist can facilitate better reporting of the transfusion-related parameters and can enable a thorough assessment of the risks and benefits of transfusion strategies adopted among VL patients in the future. Additionally, the standardisation and completeness of reporting of haematological data in VL studies before, during and after treatment, may also help in recognising the dynamics of VL clinical improvement after treatment. The individual participant data (IPD) platform hosted at IDDO is currently facilitating meta-analysis of haematological aspects of the disease, which we hope can address some of the information gaps identified in this review.^42,43^

Box 1 **Checklist of transfusion related parameters for reporting in VL efficacy studies**

**Table:** 

Item	Description
**Haematological parameters**	
	Rules used for indicating transfusion
	Haemoglobin (or haematocrit) level used for indicating transfusion
	Information regarding blood/blood products transfused: whole blood, plasma, platelets
	Units of transfused blood/blood products/transfusion volume
	Time point of transfusion: before treatment, during treatment (in days) or during the follow-up phase (in days or weeks). Report exact day if possible
	Reason for transfusion: severe anaemia, acute blood loss, epistaxis/haemorrhage/splenic bleeding
	Minimum haemoglobin concentration required for patient enrolment into a study
	Definition of anaemia/severe anaemia
**Distinction between patients and episodes**	
	Number of patients who required transfusion
	Total number of episodes of transfusion
**Therapeutic outcomes**	
	Reports of transfusion reactions, including the nature of the transfusion reaction (if any)
	Therapeutic outcomes among patients who received a transfusion

## Conclusions

Data regarding blood transfusion remain largely unreported in VL therapeutic efficacy studies, with information available in only 16 studies published since 1980. When reported, the decision to undertake transfusion was often found to be based on the haemoglobin concentration of the patients, with only three studies incorporating additional clinical criteria. Overall, this review represents an initial step in assessing practices in the use of blood products in VL clinical studies. Reporting of transfusion episodes should be undertaken thoroughly and the research community should adopt a standardised methodology so that the true risk-benefit of transfusion practices in VL case management can be reliably assessed.

## Supplementary Material

trae018_Supplemental_File

## Data Availability

All the data used in this review are available within the supplemental files.
