# Supplementary material for: Blood transfusion in the care of patients with visceral leishmaniasis: a review of practices in therapeutic efficacy studies
Source: Trans R Soc Trop Med Hyg. 2024 May 1;118(8):481–90. doi: 10.1093/trstmh/trae018 (PMC11299541; doi:10.1093/trstmh/trae018)
Supplement: trae018_Supplemental_File [file trae018_supplemental_file.docx]

**Supplemental file 1:**

**Blood transfusion in care of patients with Visceral Leishmaniasis: a review of practices in therapeutic efficacy studies**

Prabin Dahal^a,b*^, Sauman Singh-Phulgenda^a,b^, James Wilson ^a,b^, Glaucia Cota^c^, Koert Ritmeijer^d^, Ahmed Musa^e^, Fabiana Alves^f^, Kasia Stepniewska ^a,b^, Philippe J Guerin ^a,b*^

^a^ Infectious Diseases Data Observatory (IDDO), OX3 7LG, Oxford, UK

^b^ Centre for Tropical Medicine and Global Health, Nuffield Department of Medicine,

University of Oxford, OX3 7LG, Oxford, UK

^c^ Instituto René Rachou (IRR), Fiocruz, Minas Gerais, Brazil

^d^ Médecins Sans Frontières, Amsterdam, Netherlands

^e^ Institute of Endemic Diseases, University of Khartoum, Khartoum 11111, Sudan

^f^ Drugs for Neglected Diseases initiative, CH1202, Geneva, Switzerland

Contents

[Supplemental Table 1: List of studies indexed in IDDO VL clinical trials library 2](#_Toc156937010)

[Supplemental Table 2: Description of studies included in the review 9](#_Toc156937011)

[Supplemental Table 3: Risk of bias in randomised studies 10](#_Toc156937012)

[Supplemental Table 4: Risk of bias in non-randomised studies 11](#_Toc156937013)

[References of the 160 studies in IDDO’s systematic review library 13](#_Toc156937014)

## Supplemental Table 1: List of studies indexed in IDDO VL clinical trials library (n=160 studies)

Further details of the studies are available from: <https://www.iddo.org/vlSurveyor/#0>

| **IDDO ID** | **Author year** | **PUBMED ID** | **Region** | **Eligible age range** | **Pregnancy** | **Minimum haemoglobin  concentration for inclusion** | **Prothrombin time  (above control values)** | **Minimum WBC count** | **Minimum platelets  concentration** |
| --- | --- | --- | --- | --- | --- | --- | --- | --- | --- |
| 1 | Ritmeijer-2006^1^ | 16804852 | Eastern Africa | ≥15y | Excluded | Not mentioned | Not mentioned | Not mentioned | Not mentioned |
| 2 | Thakur-2001a^2^ | 11137652 | India Subcontinent | >12y | Excluded | 5 | Not mentioned | Not mentioned | Not mentioned |
| 3 | Laguna-2003^3^ | 12888588 | Mediterranean | >18y | Excluded | Not mentioned | Activity < 40% (excluded) | Not mentioned | Not mentioned |
| 4 | Giri-1993^4^ | 8409490 | India Subcontinent | all ages | Included | Not mentioned | Not mentioned | Not mentioned | Not mentioned |
| 5 | Sundar-2011b^5^ | 21255828 | India Subcontinent | 5-60y | Excluded | 5 | >5s | WBC <1,000/μL | <40,000/μL |
| 6 | Thakur-1994b^6^ | 7886778 | India Subcontinent | ≥18y | Not mentioned | Not mentioned | Not mentioned | Not mentioned | Not mentioned |
| 7 | Wali-1997^7^ | 9282505 | India Subcontinent | >12y | Not mentioned | Not mentioned | Not mentioned | Not mentioned | Not mentioned |
| 8 | Gaeta-2000^8^ | 11200380 | Mediterranean | >18y | Not mentioned | Not mentioned | Not mentioned | Not mentioned | Not mentioned |
| 9 | Berhe-1999^9^ | 10513651 | Eastern Africa | Not defined | Not mentioned | Not mentioned | Not mentioned | Not mentioned | Not mentioned |
| 10 | Jha-1999^10^ | 10588964 | India Subcontinent | 12-50y | Excluded | 6.5 | Not mentioned | WBC <2,000/μL | <75,000/μL |
| 11 | Sundar-2008b^11^ | 18781879 | India Subcontinent | ≥12y | Excluded | 3.5 | >15s | granulocytes <1,000/µl | <40,000/μL |
| 12 | Sundar-2002^12^ | 12456849 | India Subcontinent | ≥12y | Excluded | 6 | Not mentioned | WBC <1,000/μL | <50,000/μL |
| 13 | Sundar-1999a^13^ | 10707104 | India Subcontinent | 14-65y | Excluded | 6 | >4s | granulocytes <2,000/µl | <50,000/μL |
| 14 | Sundar-2011c^14^ | 21633025 | India Subcontinent | 16-50y | Excluded | Not mentioned | Not mentioned | Not mentioned | Not mentioned |
| 15 | Sundar-2009a^15^ | 19407109 | India Subcontinent | 18-65y | Excluded | 6 | >5s | granulocytes <1,000/µl | <50,000/μL |
| 16 | Sundar-2008a^16^ | 18664241 | India Subcontinent | 12-65y | Excluded | 6 | >5s | granulocytes <1,000/µl | <50,000/μL |
| 17 | Sundar-2000c^17^ | 11049800 | India Subcontinent | 12-65y | Excluded | 6 | >5s | WBC <2,000/μL | <50,000/μL |
| 18 | Laguna-1999^18^ | 10397536 | Mediterranean | >18y | Excluded | Not mentioned | >20s | Not mentioned | Not mentioned |
| 19 | Russo-1996^19^ | 8708370 | Multi-Regional | >25y | Not mentioned | Not mentioned | Not mentioned | Not mentioned | Not mentioned |
| 20 | Davidson-1994^20^ | 7811891 | Mediterranean | Not defined | Not mentioned | Not mentioned | Not mentioned | Not mentioned | Not mentioned |
| 21 | Thakur-2004a^21^ | 15035723 | India Subcontinent | >6y | Not mentioned | 6 | Not mentioned | Not mentioned | Not mentioned |
| 22 | Chulay-1983^22^ | 6309993 | Eastern Africa | all ages | Not mentioned | Not mentioned | Not mentioned | Not mentioned | Not mentioned |
| 23 | Das-2009^23^ | 19436614 | India Subcontinent | 6-60y | Excluded | 6 | Not mentioned | Not mentioned | <50,000/μL |
| 24 | Wasunna-2005^24^ | 16282296 | Eastern Africa | 5-65y | Excluded | Not mentioned | Not mentioned | Not mentioned | Not mentioned |
| 25 | Thakur-2000a^25^ | 11127250 | India Subcontinent | 6-50y | Excluded | 5 | Not mentioned | WBC <2,000/μL | <80,000/µl |
| 26 | Karki-1998^26^ | 9740292 | India Subcontinent | Not defined | Excluded | Not mentioned | Not mentioned | Not mentioned | Not mentioned |
| 27 | Jha-2005^27^ | 16354802 | India Subcontinent | 5-65y | Excluded | Not mentioned | Not mentioned | Not mentioned | Not mentioned |
| 28 | Sundar-2011a^28^ | 21129762 | India Subcontinent | 2-65y | Excluded | Not mentioned | Not mentioned | Not mentioned | Not mentioned |
| 29 | Thakur-1995^29^ | 7778155 | India Subcontinent | Not defined | Not mentioned | Not mentioned | Not mentioned | Not mentioned | Not mentioned |
| 30 | Sundar-2006^30^ | 16447104 | India Subcontinent | all ages | Excluded | 3.5 | >15s | granulocytes <1,000/µl | <40,000/μL |
| 31 | Thakur-1999^31^ | 10492770 | India Subcontinent | Not defined | Included | Not mentioned | Not mentioned | Not mentioned | Not mentioned |
| 32 | Thakur-1993c^32^ | 8477209 | India Subcontinent | Not defined | Not mentioned | 3 | Not mentioned | Not mentioned | Not mentioned |
| 33 | Sundar-2004^33^ | 14727208 | India Subcontinent | Not defined | Excluded | 3 | >5s | granulocytes <1,000/µl | <40,000/μL |
| 34 | Sundar-2007b^34^ | 17682988 | India Subcontinent | 2-65y | Excluded | 3.5 | >5s | WBC <1,000/μL | <40,000/μL |
| 35 | Thakur-1994c^35^ | 7979623 | India Subcontinent | 1-40y | Not mentioned | 5 | Not mentioned | Not mentioned | Not mentioned |
| 36 | Moore-2001^36^ | 11417033 | Eastern Africa | all ages | Excluded | Not mentioned | Not mentioned | Not mentioned | Not mentioned |
| 37 | Thakur-1996a^37^ | 8707361 | India Subcontinent | Not defined | Excluded | 5 | Not mentioned | Not mentioned | Not mentioned |
| 38 | Thakur-1984b^38^ | 6322906 | India Subcontinent | Not defined | Not mentioned | 3 | Not mentioned | Not mentioned | Not mentioned |
| 39 | Thakur-1991b^39^ | 1659239 | India Subcontinent | Not defined | Not mentioned | 3 | Not mentioned | Not mentioned | Not mentioned |
| 40 | Thakur-2008^40^ | 18765878 | India Subcontinent | Not defined | Not mentioned | 5 | Not mentioned | Not mentioned | Not mentioned |
| 41 | Anabwani-1983^41^ | 6130246 | Eastern Africa | Not defined | Not mentioned | Not mentioned | Not mentioned | Not mentioned | Not mentioned |
| 42 | Sundar-1996^42^ | 8627049 | India Subcontinent | 5-65y | Excluded | 6 | >4s | granulocytes <1,000/µl | <50,000/μL |
| 43 | Thakur-1994a^43^ | 7867139 | India Subcontinent | Not defined | Not mentioned | 3 | Not mentioned | Not mentioned | Not mentioned |
| 44 | Thakur-1998b^44^ | 9797829 | India Subcontinent | 6-60y | Excluded | Not mentioned | Not mentioned | Not mentioned | Not mentioned |
| 45 | Bodhe-1999^45^ | 10492769 | India Subcontinent | all ages | Excluded | Not mentioned | Not mentioned | Not mentioned | Not mentioned |
| 46 | Freire-1997^46^ | 11105143 | South America | 3-60y | Not mentioned | 5 | Not mentioned | WBC <2,000/μL | <40,000/μL |
| 47 | Sundar-2012^47^ | 22573856 | India Subcontinent | 6-70y | Excluded | 5 | >5 | granulocytes <1,000/µl | <40,000/μL |
| 48 | Seaman-1993^48^ | 8394861 | Eastern Africa | Not defined | Included | Not mentioned | Not mentioned | Not mentioned | Not mentioned |
| 49 | Thakur-2004b^49^ | 15489554 | India Subcontinent | Not defined | Not mentioned | Not mentioned | Not mentioned | Not mentioned | Not mentioned |
| 50 | Ritmeijer-2001^50^ | 11816442 | Eastern Africa | Not defined | Not mentioned | Not mentioned | Not mentioned | Not mentioned | Not mentioned |
| 51 | Thakur-1991a^51^ | 1649790 | India Subcontinent | Not defined | Not mentioned | 3 | Not mentioned | Not mentioned | Not mentioned |
| 52 | Jha-1983^52^ | 6868096 | India Subcontinent | Not defined | Not mentioned | Not mentioned | Not mentioned | Not mentioned | Not mentioned |
| 53 | Sundar-2000b^53^ | 11049798 | India Subcontinent | all ages | Not mentioned | Not mentioned | >5s | Not mentioned | Not mentioned |
| 54 | Hailu-2010^54^ | 21049059 | Eastern Africa | 4-60y | Excluded | 5 | Not mentioned | WBC <1,000/μL | <40,000/μL |
| 55 | Thakur-2010^55^ | 21036834 | India Subcontinent | 5-55y | Not mentioned | 5 | Not mentioned | WBC <1,000/μL | <60,000/μL |
| 56 | Rijal-2013^56^ | 23425958 | India Subcontinent | >2y | Excluded | Not mentioned | Not mentioned | Not mentioned | Not mentioned |
| 57 | Sundar-2007a^57^ | 17582067 | India Subcontinent | 5-55y | Excluded | 5 | >5s | WBC <1,000/μL | <50,000/μL |
| 58 | Nayakundi-1994^58^ | 7828500 | Eastern Africa | Not defined | Not mentioned | Not mentioned | Not mentioned | Not mentioned | Not mentioned |
| 59 | Davidson-1994b^59^ | 8153291 | Mediterranean | >0.6y | Excluded | Not mentioned | Not mentioned | Not mentioned | Not mentioned |
| 60 | Seaman-1995^60^ | 7578729 | Eastern Africa | Not defined | Not mentioned | Not mentioned | Not mentioned | Not mentioned | Not mentioned |
| 61 | Sundar-2002a^61^ | 12135284 | India Subcontinent | all ages | Excluded | Not mentioned | Not mentioned | Not mentioned | Not mentioned |
| 62 | Thakur-2001b^62^ | 11280166 | India Subcontinent | >4y | Not mentioned | 5 | Not mentioned | Not mentioned | Not mentioned |
| 63 | Musa-2010^63^ | 21049063 | Eastern Africa | 4-60y | Excluded | 5 | Not mentioned | WBC <1,000/μL | <40,000/μL |
| 64 | Sherwood-1994^64^ | 7888530 | Eastern Africa | 10-50y | Excluded | 5 | Not mentioned | WBC <1,000/μL | <30,000/μL |
| 65 | Dietze-2001^65^ | 11791957 | South America | 6-50y | Excluded | 5 | Not mentioned | WBC <1,000/μL | <30,000/μL |
| 66 | Rahman-2011^66^ | 21734127 | India Subcontinent | 2-65y | Excluded | 6 | Not mentioned | Not mentioned | Not mentioned |
| 67 | Sinha-2011^67^ | 22174722 | India Subcontinent | 2-55y | Included | Not mentioned | Not mentioned | Not mentioned | Not mentioned |
| 68 | Bhattacharya-2007^68^ | 17624846 | India Subcontinent | 2-65y | Excluded | 4 | Not mentioned | WBC <1,000/μL | Not mentioned |
| 69 | Jha-1998a^69^ | 9583927 | India Subcontinent | 6-50y | Excluded | 5 | Not mentioned | WBC <2,000/μL | <80,000/µl |
| 70 | Thakur-1988^70^ | 2840172 | India Subcontinent | Not defined | Not mentioned | 3 | Not mentioned | Not mentioned | Not mentioned |
| 71 | Mueller-2008^71^ | 18186974 | Eastern Africa | Not defined | Not mentioned | Not mentioned | Not mentioned | Not mentioned | Not mentioned |
| 72 | Khalil-2014^72^ | 24454970 | Eastern Africa | >4y | Excluded | 4 | Not mentioned | Not mentioned | <40,000/μL |
| 73 | Davidson-1996^73^ | 8783690 | Multi-Regional | Not defined | Excluded | Not mentioned | Not mentioned | Not mentioned | Not mentioned |
| 74 | Sundar-2009b^74^ | 19663597 | India Subcontinent | 5-55y | Excluded | 4 | >5s | granulocytes <1,000/µl | <40,000/μL |
| 75 | Sundar-2000a^75^ | 10897369 | India Subcontinent | 5-65y | Excluded | 6 | >4s | granulocytes <1,000/µl | <50,000/μL |
| 76 | Sundar-1997^76^ | 9230003 | India Subcontinent | 5-65y | Excluded | 6 | >4s | granulocytes <1,000/µl | <5,000/µL |
| 77 | Sundar-2003b^77^ | 12955641 | India Subcontinent | all ages | Excluded | Not mentioned | Not mentioned | Not mentioned | Not mentioned |
| 78 | Sundar-2010^78^ | 20147716 | India Subcontinent | 2-65y | Not mentioned | 3.5 | >4s | WBC <750/µL | <40,000/μL |
| 79 | Musa-2012^79^ | 22724029 | Eastern Africa | 4-60y | Excluded | 5 | Not mentioned | WBC <1,000/μL | <40,000/μL |
| 80 | Zijlstra-1993^80^ | 8236402 | Eastern Africa | Not defined | Not mentioned | Not mentioned | Not mentioned | Not mentioned | Not mentioned |
| 81 | Sundar-1998c^81^ | 9924533 | India Subcontinent | 5-65y | Excluded | 6 | >4s | granulocytes <1,000/µl | <50,000/μL |
| 82 | Dietze-1993^82^ | 8110956 | South America | Not defined | Not mentioned | Not mentioned | Not mentioned | Not mentioned | Not mentioned |
| 83 | Sundar-2001^83^ | 11520836 | India Subcontinent | all ages | Excluded | 4 | Not mentioned | granulocytes <1,000/µl | <40,000/μL |
| 84 | Dietze-1995^84^ | 7660446 | South America | Not defined | Not mentioned | Not mentioned | Not mentioned | Not mentioned | Not mentioned |
| 85 | Chunge-1990^85^ | 2167522 | Eastern Africa | >3y | Excluded | 4 | Abnormal PT | Not mentioned | Not mentioned |
| 86 | Thakur-2000b^86^ | 11127251 | India Subcontinent | 6-50y | Excluded | 5 | Not mentioned | WBC <2,000/μL | <80,000/µl |
| 87 | Thakur-1992b^87^ | 1337634 | India Subcontinent | Not defined | Not mentioned | Not mentioned | Not mentioned | Not mentioned | Not mentioned |
| 88 | Sundar-1998b^88^ | 9851383 | India Subcontinent | >14y | Excluded | 6 | >4s | granulocytes <1,000/µl | <50,000/μL |
| 89 | Jha-1995^89^ | 7611561 | India Subcontinent | Not defined | Not mentioned | 7 | Not mentioned | Not mentioned | Not mentioned |
| 90 | Mondal-2010^90^ | 20668544 | India Subcontinent | all ages | Excluded | Not mentioned | Not mentioned | Not mentioned | Not mentioned |
| 91 | Veeken-2000^91^ | 10886792 | Eastern Africa | Not defined | Not mentioned | Not mentioned | Not mentioned | Not mentioned | Not mentioned |
| 92 | Mishra-1992^92^ | 1359322 | India Subcontinent | Not defined | Not mentioned | Not mentioned | Not mentioned | Not mentioned | Not mentioned |
| 93 | Mishra-1994^93^ | 7983993 | India Subcontinent | Not defined | Not mentioned | Not mentioned | Not mentioned | Not mentioned | Not mentioned |
| 94 | Rijal-2010^94^ | 19726065 | India Subcontinent | Not defined | Not mentioned | Not mentioned | Not mentioned | Not mentioned | Not mentioned |
| 95 | Thakur-1996b^95^ | 8758093 | India Subcontinent | >18y | Excluded | 5 | Not mentioned | WBC <2,000/μL | <4,000/µL |
| 96 | Berman-1998^96^ | 9615494 | Multi-Regional | Not defined | Not mentioned | Not mentioned | Not mentioned | Not mentioned | Not mentioned |
| 97 | Patra-2012^97^ | 23087513 | India Subcontinent | Not defined | Excluded | 6 | Not mentioned | WBC <1,000/μL | <50,000/μL |
| 98 | Thakur-1993b^98^ | 8406644 | India Subcontinent | Not defined | Not mentioned | 3 | Not mentioned | Not mentioned | Not mentioned |
| 99 | Mishra-1985^99^ | 3935209 | India Subcontinent | Not defined | Not mentioned | Not mentioned | Not mentioned | Not mentioned | Not mentioned |
| 100 | Figueras Nadal-2003^100^ | 14636517 | Mediterranean | 0-14y | Not mentioned | Not mentioned | Not mentioned | Not mentioned | Not mentioned |
| 101 | Singh-2010^101^ | 20065047 | India Subcontinent | 1-14y | Not mentioned | Not mentioned | Not mentioned | granulocytes <1,000/µl | <40,000/μL |
| 102 | Castagnola-1996^102^ | 8758092 | Mediterranean | Less than 15y | Excluded | Not mentioned | Not mentioned | Not mentioned | Not mentioned |
| 103 | Thakur-1993^103^ | 8244483 | India Subcontinent | Less than 15y | Not mentioned | 4 | Not mentioned | Not mentioned | Not mentioned |
| 104 | Bhattacharya-2004^104^ | 14699453 | India Subcontinent | 2-11y | Excluded | 6 | Not mentioned | WBC <1,000/μL | <50,000/μL |
| 105 | Singh-2006^105^ | 17202605 | India Subcontinent | 1-14y | Not mentioned | Not mentioned | Not mentioned | granulocytes <1,000/µl | <40,000/μL |
| 106 | Singh-2006^106^ | 17202633 | India Subcontinent | 1-14y | Not mentioned | Not mentioned | Not mentioned | granulocytes <1,000/µl | <40,000/μL |
| 107 | Sundar-2003^107^ | 12792385 | India Subcontinent | 2-11y | Not mentioned | 6 | Not mentioned | WBC <2,000/nL | <50,000/nl |
| 108 | Karimi-1998^108^ | Not indexed | Central Asia | children | Not mentioned | Not mentioned | Not mentioned | Not mentioned | Not mentioned |
| 109 | di Martino-1997^109^ | 9290615 | Mediterranean | <14y | Excluded | Not mentioned | Not mentioned | Not mentioned | Not mentioned |
| 110 | Syriopoulou-2003^110^ | 12594635 | Mediterranean | <14y | Not mentioned | Not mentioned | Not mentioned | Not mentioned | Not mentioned |
| 111 | Haidar-2001^111^ | 11426243 | Central Asia | <12y | Not mentioned | Not mentioned | Not mentioned | Not mentioned | Not mentioned |
| 112 | Tobaigy-1986^112^ | 3014837 | Central Asia | Not defined | Not mentioned | Not mentioned | Not mentioned | Not mentioned | Not mentioned |
| 113 | Sahay-1996^113^ | 8979611 | India Subcontinent | Not defined | Not mentioned | 7 | Not mentioned | Not mentioned | Not mentioned |
| 114 | Mishra-1991^114^ | 1673012 | India Subcontinent | Not defined | Not mentioned | Not mentioned | Not mentioned | Not mentioned | Not mentioned |
| 115 | Das-2005^115^ | 16130613 | India Subcontinent | Not defined | Not mentioned | Not mentioned | Not mentioned | Not mentioned | Not mentioned |
| 116 | Chowdhury-1991^116^ | 1645929 | India Subcontinent | Not defined | Not mentioned | Not mentioned | Not mentioned | Not mentioned | Not mentioned |
| 117 | Rees-1984^117^ | 6329010 | Eastern Africa | Not defined | Included | Not mentioned | Not mentioned | Not mentioned | Not mentioned |
| 118 | Rijal-2003^118^ | 15228258 | India Subcontinent | Not defined | Not mentioned | Not mentioned | Not mentioned | Not mentioned | Not mentioned |
| 119 | Thakur-1998^119^ | 11229246 | India Subcontinent | Not defined | Included | 5 | Not mentioned | WBC <2,000/μL | <80,000/µl |
| 120 | Das-2001^120^ | 11584934 | India Subcontinent | 5-60y | Not mentioned | 4 | Not mentioned | Not mentioned | Not mentioned |
| 121 | Sundar-1999b^121^ | 10999086 | India Subcontinent | 5-65y | Excluded | 6 | >4s | granulocytes <1,000/µl | <50,000/μL |
| 122 | Jha-1991^122^ | 1938817 | India Subcontinent | Not defined | Not mentioned | Not mentioned | Not mentioned | Not mentioned | Not mentioned |
| 123 | Singh-1995^123^ | 9081958 | India Subcontinent | Not defined | Not mentioned | Not mentioned | Not mentioned | Not mentioned | Not mentioned |
| 124 | Giri-1994b^124^ | 7883660 | India Subcontinent | 18-60y | Not mentioned | Not mentioned | Not mentioned | Not mentioned | Not mentioned |
| 125 | Lal-1996^125^ | 9251373 | India Subcontinent | Not defined | Not mentioned | Not mentioned | Not mentioned | Not mentioned | Not mentioned |
| 126 | Giri-1994a^126^ | 7883659 | India Subcontinent | Adults | Not mentioned | Not mentioned | Not mentioned | Not mentioned | Not mentioned |
| 127 | Thakur-1984^127^ | 6087515 | India Subcontinent | Not defined | Not mentioned | Not mentioned | Not mentioned | Not mentioned | Not mentioned |
| 128 | Jha-1998b^128^ | 9707883 | India Subcontinent | 5-15y | Not mentioned | Not mentioned | Not mentioned | Not mentioned | Not mentioned |
| 129 | Ostyn-2014^129^ | 24941345 | India Subcontinent | Not defined | Not mentioned | Not mentioned | Not mentioned | Not mentioned | Not mentioned |
| 130 | Sinha-2010^130^ | 20682882 | India Subcontinent | ≥ 2y | Included | Not mentioned | Not mentioned | Not mentioned | Not mentioned |
| 131 | Mueller-2007^131^ | 16730363 | Eastern Africa | Not defined | Included | Not mentioned | Not mentioned | Not mentioned | Not mentioned |
| 132 | Cota-2014^132^ | 24743472 | South America | >18y | Excluded | Not mentioned | Not mentioned | Not mentioned | Not mentioned |
| 133 | Sudarshan-2011^133^ | 21609983 | India Subcontinent | 6-55y | Not mentioned | Not mentioned | Not mentioned | Not mentioned | Not mentioned |
| 134 | Sundar-1998a^134^ | 9675482 | India Subcontinent | 5-45y | Not mentioned | Not mentioned | Not mentioned | Not mentioned | Not mentioned |
| 135 | Adam-2009^135^ | 19766208 | Eastern Africa | Not defined | Included | Not mentioned | Not mentioned | Not mentioned | Not mentioned |
| 136 | Shahian-2009^136^ | 19478699 | Central Asia | Not defined | Excluded | Not mentioned | Not mentioned | Not mentioned | Not mentioned |
| 137 | Villanueva-2000^137^ | 11117648 | Multi-Regional | Not defined | Not mentioned | Not mentioned | Not mentioned | Not mentioned | Not mentioned |
| 138 | Thakur-1992a^138^ | 1323160 | India Subcontinent | >12y | Not mentioned | Not mentioned | Not mentioned | Not mentioned | Not mentioned |
| 139 | Thakur-1998a^139^ | 9654264 | India Subcontinent | Not defined | Not mentioned | Not mentioned | Not mentioned | Not mentioned | Not mentioned |
| 140 | Goswami-2016^140^ | 26526926 | India Subcontinent | Not defined | Excluded | Not mentioned | Not mentioned | WBC <1,000/μL | <50,000/μL |
| 141 | Jamil-2015^141^ | 26496648 | India Subcontinent | 5-55y | Excluded | anaemic patients excluded | Not mentioned | Not mentioned | Not mentioned |
| 142 | Sundar-2015^142^ | 25510715 | India Subcontinent | 12-60y | Excluded | 5 | >5s | Not mentioned | <40,000/μL |
| 143 | Sundar-2014^143^ | 25233346 | India Subcontinent | 5-65y | Excluded | 5 | >4s | WBC <1,000/μL | <50,000/μL |
| 144 | Mondal-2014^144^ | 25104636 | India Subcontinent | >5y | Excluded | Not mentioned | Not mentioned | Not mentioned | Not mentioned |
| 145 | Rashid-1994^145^ | 7835263 | Eastern Africa | >5y | Excluded | 6 | >5s | Not mentioned | <40,000/μL |
| 147 | Sundar-2019^146^ | 31436156 | India Subcontinent | 5-65y | Not mentioned | Not mentioned | Not mentioned | Not mentioned | Not mentioned |
| 148 | Diro-2019^147^ | PMC6336227 | Eastern Africa | 18-60y | Excluded | Not mentioned | Not mentioned | Not mentioned | Not mentioned |
| 149 | Mbui-2018^148^ | 30188978 | Eastern Africa | 4-12y | Excluded | 5 | Not mentioned | WBC <1,000/mL | <40,000/mL |
| 154 | Borges-2017^149^ | 28327804 | South America | 0.5-12y | Not mentioned | Not mentioned | Not mentioned | Not mentioned | Not mentioned |
| 155 | Wasunna-2016^150^ | 27627654 | Eastern Africa | 7-60y | Excluded | 5 | Not mentioned | WBC <1,000/μL | <40,000/μL |
| 156 | Rahman-2017^151^ | 28558062 | India Subcontinent | 5-60y | Excluded | 5 | >5s | Not mentioned | Not mentioned |
| 159 | Romero-2017^152^ | 28662034 | South America | 0.5-50y | Excluded | Not mentioned | INR>2 | Not mentioned | <20,000/μL |
| 164 | Goswami-2020^153^ | 32394874 | India Subcontinent | 5-65y | Excluded | Not mentioned | Not mentioned | Not mentioned | Not mentioned |
| 165 | Alborzi-2017^154^ | 27879460 | Central Asia | Not defined | Not mentioned | Not mentioned | Not mentioned | Not mentioned | Not mentioned |
| 168 | Pandey-2017^155^ | 29016288 | India Subcontinent | <15y | Not mentioned | 5 | Not mentioned | WBC <1,000/μL | <50,000/μL |
| 169 | Pandey-2016^156^ | 27645786 | India Subcontinent | 6-70y | Excluded | 5 | >5s | granulocytes <1,000/µl | <40,000/μL |
| 170 | Kimutai-2017^157^ | PMC5315726 | Eastern Africa | Not defined | Included | Not mentioned | Not mentioned | Not mentioned | Not mentioned |
| 171 | Goyal-2018^158^ | 30346949 | India Subcontinent | >2y | Included | Not mentioned | Not mentioned | Not mentioned | Not mentioned |
| 179 | Sinha-2019^159^ | Not indexed | India Subcontinent | 2-11y | Excluded | 6 | Not mentioned | WBC <1,000/μL | <50,000/μL |
| 182 | Ekram-2021^160^ | 34789971 | India Subcontinent | 3-65y | Excluded | Not mentioned | Not mentioned | Not mentioned | Not mentioned |

## Supplemental Table 2: Description of studies included in the review (n=16 studies)

| **Author-year** | **Country** | **Total enrolled** | **Duration of illness (days)** | **Age-range of included participants** | **Inclusion of HIV co-infected patients** | **Inclusion of pregnant women** | **Drug used for treatment** |
| --- | --- | --- | --- | --- | --- | --- | --- |
| Rees-1984^117^ | Kenya | 16 | 53.2 | All ages | Unclear | Included | SSG |
| Thakur-1984^127^ | India | 750 | - | All ages | Unclear | Unclear | SSG |
| Thakur-1988^70^ | India | 371 | 166.5 | All ages (9-45y) | Unclear | Unclear | SSG |
| Thakur-1991^39^ | India | 312 | - | All ages (2-60y) | Unclear | Unclear | Pentamidine; Pentamidine + SSG |
| Dietze-1993^82^ | Brazil | 20 | 90 | All ages (1-57y) | Unclear | Unclear | ABCD |
| Thakur-1993^103^ | India | 50 | - | Children (<15y) | Unclear | Unclear | Amphotericin B |
| Berhe-1999^9^ | Ethiopia | 23 | - | Adults (20-47y) | Included | Unclear | PA |
| Thakur-1999^31^ | India | 938 | - | All ages (0.8-80y) | Unclear | Included | AMBd |
| Moore-2001^36^ | Kenya | 102 | - | All ages (2-40y) | Unclear | Excluded | SSG |
| Haidar-2001^111^ | Yemen | 32 | 168.7 | Children (<12y) | Unclear | Unclear | SSG |
| Mueller-2008^71^ | Uganda | 371 | 28-53 | All ages | Unclear | Unclear | AMBd; PA |
| Das-2009^23^ | India | 82 | - | All ages (6-60y) | Excluded | Excluded | AMBd; Pentamidine |
| Adam-2009^135^ | Sudan | 42 | - | All ages (29.7±14.5 y) | Excluded | Included | SSG |
| Thakur-2010^55^ | India | 230 | - | All ages (5-55y) | Excluded | Unclear | Amphotericin B |
| Cota-2014^132^ (HIV negative) | Brazil | 46 | 60 | All ages (37.1 ± SD: 14.0y) | Excluded | Excluded | PA; AMBd; L-AmB |
| Cota-2014^132^ (HIV positive) | Brazil | 44 | 60 | All ages (41.0 ± SD: 10.9) | Included | Excluded | PA; AMBd; L-AmB |
| Mbui-2018^148^ | Kenya, Uganda | 30 | - | Children (4-12y) | Excluded | Excluded | Miltefosine |

SSG=Sodium stibogluconate; PA=Pentavalent antimony; AMB=Amphotericin B; AMBd=Amphotericin B deoxycholate; ABCD=Amphotericin B colloidal dispersion; SD=standard deviation

## Supplemental Table 3: Risk of bias in randomised studies

| **IDDO ID** | **Author-year** | **Blinding details** | **Study conduct details** | **Domain** | | | | | | |
| --- | --- | --- | --- | --- | --- | --- | --- | --- | --- | --- |
|  |  |  |  | **Random sequence  generation** | **Allocation  concealment** | **Blinding of**  **participants  and personnel** | **Blinding of  outcome assessment** | **Incomplete  outcome data addressed** | **Selective  reporting** |  |
| 23 | Das-2009^23^ | Open | Treatment allocation was done by the biostatistician of the institute, who performed the allocation sequence using random number tables and accordingly assigned the test and control group. The patients were sent to the indoor ward for further treatment with their allotted drug. Both groups of patients were treated after the hospitalization in RMRIMS indoor ward. Every patient completed the full course of assigned treatment. | Low | Unclear | High | High | Low | Low |  |
| 39 | Thakur-1991b^39^ | Unclear | Patients were randomly allocated to three treatment groups. | Unclear | Unclear | Unclear | Unclear | Low | Low |  |
| 55 | Thakur-2010^55^ | Open | This study was conducted as an open-label, randomized trial of 230 patients at Balaji Utthan Sansthan, Patna. The study staff who treated the patients opened consecutively numbered envelopes containing the treatment assignment after eligible patients fulfilled the entry criteria. Clinicians who provided treatment were not blinded to the treatment given. | Unclear | Low | High | Unclear | Low | Low |  |
| 70 | Thakur-1988^70^ | Unclear | The patients were randomly allocated to six treatment groups. | Unclear | Unclear | Unclear | Low | Low | Low |  |

## Supplemental Table 4: Risk of bias in non-randomised studies

| **IDDO ID** | **Author-year** | **Description of study design and conduct** | **Bias due to confounding (Imbalances in baseline distribution)** | **Bias in selection  of participants** | **Bias in intervention  classification** | **Missing  outcome data** | **Bias in  outcome assessment** | **Selective  outcome reporting** | **Treatment**  **Blinding** |
| --- | --- | --- | --- | --- | --- | --- | --- | --- | --- |
| 9 | Berhe-1999^9^ | Twenty-three consecutive HIV-VL patients with no other obvious concurrent infectious diseases were recruited from an ongoing VL-HIV co-infection study. | Not applicable;  single-armed trial | Low/moderate  (consecutive patient case series) | - | Unclear | Low | Unclear | Unclear |
| 31 | Thakur-1999^31^ | Confirmed cases of visceral leishmaniasis were included in this study of all consecutive cases coming for treatment between 1 January and 31 December 1997 at the Kala-azar Research Centre of Balaji Utthan Sansthan, Patna. | Not applicable;  single-armed trial | Low/moderate  (consecutive patient case series) | - | Low | Low | Low | Unclear |
| 36 | Moore-2001^36^ | The allocation of patients to treatment groups was alternate and not random, and the hospital staff were not blinded to the treatment given. However, the slides of splenic aspirates were read ‘‘blind’’, and the main outcome measures (death, initial cure, or definitive cure) are unlikely to have been affected by a knowledge of the treatment received. | Moderate/High (Table 1 of the manuscript) | Low/moderate (Table 1 of the manuscript) | Low | High | Low | Low | Open |
| 71 | Mueller-2008^71^ | Between September 2003 and April 2004, the supply of antimonial drugs to Amudat Hospital, in north–eastern Uganda, was interrupted and all cases of visceral leishmaniasis presenting at the hospital could only be treated with amphotericin B deoxycholate (AmB). For comparison with the results of the AmB treatment, an historical cohort, of all the patients diagnosed with first-time VL when they presented at the Amudat Hospital between September 2002 and April 2003, was selected. The patients in this cohort had all been treated with intramuscular injections of SbV, given at 20 mg/kg/day (without an upper limit) for 30 days. | Moderate/High (Table 1 of the manuscript gives reasonably similar distribution of the baseline covariates) | Low/moderate | Low | Low | Low | Low | Unclear |
| 82 | Dietze-1993^82^ | This study was an open-label, phase 1/2 clinical trial of the efficacy and toxicity of Amphocil. Two cohorts, each consisting of 10 consecutive patients with kala-azar, were treated with Amphocil at a dosage of 2 mg kg/day. | Moderate/High (Table 1 of the manuscript gives reasonably similar distribution of the baseline covariates) | Low/moderate | Low | Low | Low | Low | Open |
| 103 | Thakur-1993^103^ | Study in fifty children suffering from multiple drug resistant kala-azar, with classical features of severe kala-azar | Not applicable;  single-armed trial | High  (drug resistant VL cases enrolled in the study) | - | Low | Low | Low | Unclear |
| 111 | Haidar-2001^111^ | A prospective hospital-based study in children of 12 years of age or less. | Not applicable;  single-armed trial | Low/moderate | - | Unclear | Low | Unclear | Unclear |
| 117 | Rees-1984^117^ | Sixteen consecutive new patients with kala-azar seen by us between September 1978 and February 1979 at the Kenyatta National Hospital, Nairobi, were studied. | Not applicable;  single-armed trial | Low/moderate  (consecutive patient case series) | - | Unclear | Low | Low | Unclear |
| 127 | Thakur-1984^127^ | Data from outbreak setting. | Not applicable;  single-armed trial | Low/moderate as all data from the outbreak is reported | Low | Low | Low | Low | Unclear |
| 132 | Cota-2014^132^ | The study was conducted at a reference centre for infectious diseases in Brazil. All patients with suspected VL were evaluated in an ongoing cohort study. | Unclear  (table 1 stratified by HIV status) | Unclear | Low | Low | Low | Low | Unclear |
| 135 | Adam-2009^135^ | Prospective cohort study. | Not applicable;  single-armed trial | Low/moderate | - | Unclear | Low | Moderate | Unclear |
| 149 | Mbui-2018^148^ | Open-label clinical trial. | Not applicable;  single-armed trial | Low/moderate | - | Low | Low | Low | Open |

## References of the 160 studies in IDDO’s systematic review library

1. Ritmeijer K, Dejenie A, Assefa Y, et al. A Comparison of Miltefosine and Sodium Stibogluconate for Treatment of Visceral Leishmaniasis in an Ethiopian Population with High Prevalence of HIV Infection. *Clin Infect Dis*. 2006;43(3):357-364. doi:10.1086/505217

2. Thakur CP. A single high dose treatment of kala-azar with Ambisome (amphotericin B lipid complex): a pilot study. *Int J Antimicrob Agents*. 2001;17:67-70.

3. Laguna F, Videla S, Jiménez-Mejías ME, et al. Amphotericin B lipid complex versus meglumine antimoniate in the treatment of visceral leishmaniasis in patients infected with HIV: A randomized pilot study. *J Antimicrob Chemother*. 2003;52(3):464-468. doi:10.1093/jac/dkg356

4. Giri O. Amphotericin B therapy in kala azar.pdf. *J Indian Med Assoc*. 1993;91(4):91-93.

5. Sundar S, Sinha PK, Rai M, et al. Comparison of short-course multidrug treatment with standard therapy for visceral leishmaniasis in India: An open-label, non-inferiority, randomised controlled trial. *Lancet*. 2011;377(9764):477-486. doi:10.1016/S0140-6736(10)62050-8

6. Thakur CP. Comparison of glucose versus fat emulsion in the preparation of amphotericin B for use in kala-azar. *Trans R Soc Trop Med Hyg*. 1994;88(6):698-699. doi:10.1016/0035-9203(94)90236-4

7. Wali JP, Aggarwal P, Nandy A, et al. Efficacy of sodium antimony gluconate and ketoconazole in the treatment of kala-azar - A comparative study. *J Commun Dis*. 1997;29(2):73-83.

8. Gaeta GB, Maisto A, Caprio DDI, et al. Efficacy of Amphotericin B Colloidal Dispersion in the Treatment of Mediterranean Visceral Leishmaniasis in Immunocompetent Adult Patients. *Scand J Infect Dis*. Published online 2000:675-677.

9. Berhe N, Wolday D, Hailu A, et al. HIV viral load and response to antileishmanial chemotherapy in co-infected patients. *AIDS*. 1999;13(14):1921-1925. doi:10.1097/00002030-199910010-00015

10. Jha TK, Sundar S, Thakur CP, et al. Miltefosine, an Oral Agent, for the Treatment of Indian Visceral Leishmaniasis. *N Engl J Med*. 1999;341(24):1795-1800. doi:10.1056/NEJM199912093412403

11. Sundar S, Rai M, Chakravarty J, et al. New Treatment Approach in Indian Visceral Leishmaniasis: Single‐Dose Liposomal Amphotericin B Followed by Short‐Course Oral Miltefosine. *Clin Infect Dis*. 2008;47(8):1000-1006. doi:10.1086/591972

12. Sundar S, Jha T, Thakur C, et al. ORAL MILTEFOSINE FOR INDIAN VISCERAL LEISHMANIASIS. *N Engl J Med*. 2002;347(22):1739-1746. doi:10.1056/NEJMoa012295

13. Sundar S, Gupta L, Makharia M, et al. Oral treatment of visceral leishmaniasis with miltefosine. *Ann Trop Med Parasitol*. 1999;93(6):589-597.

14. Sundar S, Sinha PK, Dixon SA, et al. Pharmacokinetics of oral sitamaquine taken with or without food and safety and efficacy for treatment of visceral leishmaniais: A randomized study in Bihar, India. *Am J Trop Med Hyg*. 2011;84(6):892-900. doi:10.4269/ajtmh.2011.10-0409

15. Sundar S, Singh A, Agarwal D, Rai M, Agrawal N, Chakravarty J. Safety and efficacy of high-dose infusions of a preformed amphotericin B fat emulsion for treatment of indian visceral leishmaniasis. *Am J Trop Med Hyg*. 2009;80(5):700-703. doi:80/5/700 [pii]

16. Sundar S, Chakravarty J, Agarwal D, Shah A, Agrawal N, Rai M. Safety of a pre-formulated amphotericin B lipid emulsion for the treatment of Indian Kala-azar. *Trop Med Int Heal*. 2008;13(9):1208-1212. doi:10.1111/j.1365-3156.2008.02128.x

17. Sundar S, Makharia A, More DK, et al. Short-course of oral miltefosine for treatment of visceral leishmaniasis. *Clin Infect Dis*. 2000;31(4):1110-1113. doi:10.1086/318122

18. Laguna F, Pulido F, Salas A, et al. Treatment of visceral leishmaniasis in HIV-infected patients: a randomized trial comparing meglumine antimoniate with amphotericin B. *Aids*. 1999;(November 1998).

19. Russo R, Nigro LC, Minniti S, et al. Visceral leishmaniasis in HIV infected patients: treatment with high dose liposomal amphotericin B (AmBisome). *J Infect*. 1996;32(2):133-137. http://www.ncbi.nlm.nih.gov/pubmed/8708370

20. Davidson R, Russo R. Relapse of Visceral Leishmaniasis in Patients Who Were Coinfected with Human Immunodeficiency Virus and Who Received Treatment with Liposomal Amphotericin B. *Clin Infect Dis*. 1994;19(Sep):560.

21. Thakur CP, Narayan S. A comparative evaluation of amphotericin B and sodium antimony gluconate, as first-line drugs in the treatment of Indian visceral leishmaniasis. *Ann Trop Med Parasitol*. 2004;98(2):129-138. doi:10.1179/000349804225003154

22. Chulay JD, Bhatt SM, Muigai R, et al. A Comparison of 3 Dosage Regimens of Sodium Stibogluconate in the Treatment of Visceral Leishmaniasis in Kenya. *J Infect Dis*. 1983;148(1):148-155.

23. Das VNR, Siddiqui NA, Pandey K, et al. A controlled, randomized nonblinded clinical trial to assess the efficacy of amphotericin B deoxycholate as compared to pentamidine for the treatment of antimony unresponsive visceral leishmaniasis cases in Bihar, India. *Ther Clin Risk Manag*. 2009;5(1):117-124. doi:10.2147/TCRM.S3581

24. Wasunna MK, Rashid JR, Mbui J, et al. A phase II dose-increasing study of sitamaquine for the treatment of visceral leishmaniasis in Kenya. *Am J Trop Med Hyg*. 2005;73(5):871-876. doi:73/5/871 [pii]

25. Thakur CP, Kanyok TP, Pandey AK, et al. A prospective randomized, comparative, open-label trial of the safety and efficacy of paromomycin (aminosidine) plus sodium stibogluconate versus sodium stibogluconate alone for the treatment of visceral leishmaniasis. *Trans R Soc Trop Med Hyg*. 2000;94(4):429-431. doi:10.1016/S0035-9203(00)90130-5

26. Karki P, Koirala S, Parija SC, Hansdak SG, Das ML. A thirty day course of sodium stibogluconate for treatment of Kala-azar in Nepal. *Southeast Asian J Trop Med Public Health*. 1998;29(1):154-158.

27. Jha TK, Sundar S, Thakur CP, Felton JM, Sabin AJ, Horton J. A phase II dose-ranging study of sitamaquine for the treatment of visceral leishmaniasis in India. *Am J Trop Med Hyg*. 2005;73(6):1005-1011. doi:73/6/1005 [pii]

28. Sundar S, Sinha PK, Verma DK, et al. Ambisome plus miltefosine for Indian patients with kala-azar. *Trans R Soc Trop Med Hyg*. 2011;105(2):115-117. doi:10.1016/j.trstmh.2010.10.008

29. Thakur CP, Bhowmick S, Doti L, et al. Aminosidine plus sodium stibogluconate for the treatment of Indian kala-azar: a randomized dose-finding clinical trial. *Clin Lab*. 1995;89:219-223.

30. Sundar S, Mehta H, Chhabra A, et al. Amphotericin B colloidal dispersion for the treatment of Indian visceral leishmaniasis. *Clin Infect Dis*. 2006;42(5):608-613. doi:10.1086/500138

31. Thakur CP, Singh RK, Hassan SM, Kumar R, Narain S, Kumar A. Amphotericin B deoxycholate treatment of visceral leishmaniasis with newer modes of administration and precautions: A study of 938 cases. *Trans R Soc Trop Med Hyg*. 1999;93(3):319-323. doi:10.1016/S0035-9203(99)90037-8

32. Thakur CP, Sinha GP, Pandey AK, Barat D, Sinha PK. Amphotericin B in resistant kala-azar in Bihar. *Natl Med J India*. 1993;6(2):57-60.

33. Sundar S, Mehta H, Suresh a V, Singh SP, Rai M, Murray HW. Amphotericin B treatment for Indian visceral leishmaniasis: conventional versus lipid formulations. *Clin Infect Dis*. 2004;38(3):377-383. doi:10.1086/380971

34. Sundar S, Chakravarty J, Rai VK, et al. Amphotericin B Treatment for Indian Visceral Leishmaniasis: Response to 15 Daily versus Alternate-Day Infusions. *Clin Infect Dis*. 2007;45(5):556-561. doi:10.1086/520665

35. Thakur CP, Sinha GP, Barat D, Singh RK. Are incremental doses of amphotericin B required for the treatment of visceral leishmaniasis? *Ann Trop Med Parasitol*. 1994;88(4):365-370. doi:10.1080/00034983.1994.11812878

36. Moore E, O’Flaherty D, Heuvelmans H, et al. Comparison of generic and proprietary sodium stibogluconate for the treatment of visceral leishmaniasis in Kenya. *Bull World Health Organ*. 2001;79(5):388-393.

37. Thakur CP, Sinha GP, Pandey AK. Comparison of regimens of amphotericin B deoxycholate in kala-azar. *Indian J Med Res*. 1996;103(MAY):259-263.

38. Thakur CP, Kumar M, Singh SK, et al. Comparison of regimens of treatment with sodium stibogluconate in kala-azar. *Br Med J (Clin Res Ed)*. 1984;288(6421):895-897. http://www.ncbi.nlm.nih.gov/pubmed/18765878

39. Thakur CP, Kumar M, Pandey AK. Comparisons of regimens of treatment of antimony-resistant Kala-Azar patients: A randomized study. *Am J Trop Med Hyg*. 1991;45(4).

40. Thakur CP, Thakur S, Narayan S, Sinha A. Comparison of treament regimens of kala-azar based on culture and sensitivity amastigotes to sodium antimony gluconate. *Indian J Med Res*. 2008;127(6):582-588.

41. Anabwani GM, Dimiti G, Ngira JA, Bryceson ADM. Comparison of Two Dosage Schedules of Sodium Stibogluconate in the Treatment of Visceral Leishmaniasis in Kenya. *Lancet*. 1983;321(8318):210-212. doi:10.1016/S0140-6736(83)92588-6

42. Sundar S, Murray HW. Cure of antimony-unresponsive Indian visceral leishmaniasis with amphotericin B lipid complex. *J Infect Dis*. 1996;173(3):762-765. doi:10.1093/infdis/173.3.762

43. Thakur CP, Sinha GP, Pandey AK, Barat D, Singh RK. Daily versus alternate-day regimen of amphotericin B in the treatment of kala-azar: A randomized comparison. *Bull World Health Organ*. 1994;72(6):931-936.

44. Thakur CP, Sinha GP, Pandey AK, et al. Do the diminishing efficacy and increasing toxicity of sodium stibogluconate in the treatment of visceral leishmaniasis in Bihar, India, justify its continued use as a first-line drug? An observational study of 80 cases. *Ann Trop Med Parasitol*. 1998;92(5):561-569. doi:10.1080/00034983.1998.11813313

45. Bodhe P V., Kotwani RN, Kirodian BG, et al. Dose-ranging studies on liposomal amphotericin B (L-AMP-LRC-1) in the treatment of visceral leishmaniasis. *Trans R Soc Trop Med Hyg*. 1999;93(3):314-318. doi:10.1016/S0035-9203(99)90036-6

46. Freire M, Badaro F, Avelar ME, et al. Efficacy and Tolerability of Liposomal Amphotericin B (Ambisome) in the Treatment of Visceral Leishmaniasis in Brazil. *Braz J Infect Dis*. 1997;1(5):230-240.

47. Sundar S, Singh A, Rai M, et al. Efficacy of miltefosine in the treatment of visceral leishmaniasis in India after a decade of use. *Clin Infect Dis*. 2012;55(4):543-550. doi:10.1093/cid/cis474

48. Seaman J, Pryce D, Sondorp HE, Moody A, Bryceson ADM, Davidson RN. Epidemic Visceral Leishmaniasis in Sudan: A Randomized Trial of Aminosidine plus Sodium Stibogluconate versus Sodium Stibogluconate Alone. *J Infect Dis*. 1993;168(3):715-720. doi:10.1093/infdis/168.3.715

49. Thakur CP, Narayan S, Ranjan A. Epidemiological, clinical & pharmacological study of antimony-resistant visceral leishmaniasis in Bihar, India. *Indian J Med Res*. 2004;120(3):166-172. http://www.ncbi.nlm.nih.gov/pubmed/15489554

50. Ritmeijer K, Veeken H, Melaku Y, et al. Ethiopian visceral leishmaniasis: Generic and proprietary sodium stibogluconate are equivalent; HIV co-infected patients have a poor outcome. *Trans R Soc Trop Med Hyg*. 2001;95(6):668-672. doi:10.1016/S0035-9203(01)90110-5

51. Thakur CP, Kumar M, Pandey AK. Evaluation of efficacy of longer durations of therapy of fresh cases of kala-azar with sodium stibogluconate. *Indian J Med Res*. 1991;93(March):103-110.

52. Jha TK. Evaluation of diamidine compound (pentamidine isethionate) in the treatment of resistant cases of kala-azar occurring in North Bihar, India. *Trans R Soc Trop Med Hyg*. 1983;77(2):167-170. doi:10.1016/0035-9203(83)90058-5

53. Sundar S, More DK, Singh MK, et al. Failure of Pentavalent Antimony in Visceral Leishmaniasis in India: Report from the Center of the Indian Epidemic. *Clin Infect Dis*. 2000;31(4):1104-1107. doi:10.1086/318121

54. Hailu A, Musa A, Wasunna M, et al. Geographical variation in the response of visceral leishmaniasis to paromomycin in East Africa: A multicentre, open-label, randomized trial. *PLoS Negl Trop Dis*. 2010;4(10). doi:10.1371/journal.pntd.0000709

55. Thakur CP, Kumar A, Mitra DK, Roy A, Sinha AK, Ranjan A. Improving outcome of treatment of Kala-Azar by supplementation of amphotericin B with physiologic saline and potassium chloride. *Am J Trop Med Hyg*. 2010;83(5):1040-1043. doi:10.4269/ajtmh.2010.10-0255

56. Rijal S, Ostyn B, Uranw S, et al. Increasing failure of miltefosine in the treatment of kala-azar in nepal and the potential role of parasite drug resistance, reinfection, or noncompliance. *Clin Infect Dis*. 2013;56(11):1530-1538. doi:10.1093/cid/cit102

57. Sundar S, Jha TK, Thakur CP, Sinha PK, Bhattacharya SK. Injectable Paromomycin for Visceral Leishmaniasis in India. *N Engl J Med*. 2007;356(25):2571-2581.

58. Nyakundi P, Wasunna K, Rashid JR, et al. Is one year follow up justified in Kala Azar post treatment. *East Afr Med J*. 1994;71(7).

59. Davidson RN, Dimartino L, Gradoni L, et al. Liposomal Amphotericin-B (Ambisome) in Mediterranean Visceral Leishmaniasis - a Multicenter Trial. *Q J Med*. 1994;87(2):75-81.

60. Seaman J, Boer C, Wilkinson R, et al. Liposomal Amphotericin B (AmBisome) in the Treatment of Complicated Kala- Azar Under Field Conditions. *Clin Infect Dis*. 1995;21:188-193.

61. Sundar S, Jha TK, Thakur CP, Mishra M, Singh VP, Buffels R. Low-dose liposomal amphotericin B in refractory Indian visceral leishmaniasis: A multicenter study. *Am J Trop Med Hyg*. 2002;66(2):143-146. doi:10.4269/ajtmh.2002.66.143

62. Thakur CP, Ahmed S. Observations on amphotericin B treatment of kala-azar given in a rural set up in Bihar, India. *Indian J Med Res*. 2001;113(JAN.):14-18.

63. Musa AM, Younis B, Fadlalla A, et al. Paromomycin for the treatment of visceral leishmaniasis in Sudan: A randomized, open-label, dose-finding study. *PLoS Negl Trop Dis*. 2010;4(10):4-10. doi:10.1371/journal.pntd.0000855

64. Sherwood JA, Gachihi GS, Muigai RK, et al. Phase 2 efficacy trial of an oral 8-aminoquinoline (WR6026) for treatment of visceral leishmaniasis. *Clin Infect Dis*. 1994;19(6):1034-1039. http://www.ncbi.nlm.nih.gov/pubmed/7888530

65. Dietze R, Carvalho SFG, Valli LC, et al. Phase 2 trial of WR6026, an orally administered 8-aminoquinoline, in the treatment of visceral leishmaniasis caused by Leishmania chagasi. *Am J Trop Med Hyg*. 2001;65(6):685-689. doi:10.4269/ajtmh.2001.65.685

66. Rahman M, Ahmed BN, Faiz MA, et al. Phase IV trial of miltefosine in adults and children for treatment of visceral leishmaniasis (kala-azar) in Bangladesh. *Am J Trop Med Hyg*. 2011;85(1):66-69. doi:10.4269/ajtmh.2011.10-0661

67. Sinha PK, Jha TK, Thakur CP, et al. Phase 4 pharmacovigilance trial of paromomycin injection for the treatment of visceral leishmaniasis in India. *J Trop Med*. 2011;2011. doi:10.1155/2011/645203

68. Bhattacharya SK, Sinha PK, Sundar S, et al. Phase 4 Trial of Miltefosine for the Treatment of Indian Visceral Leishmaniasis. *J Infect Dis*. 2007;196(4):591-598. doi:10.1086/519690

69. Jha TK, Olliaro P, Thakur CP, et al. Randomised controlled trial of aminosidine (paromomycin) v sodium stibogluconate. *Bmj*. 1998;316(7139):1200-1205.

70. Thakur CP, Kumar M, Kumar P, Mishra BN, Pandey AK. Rationalisation of regimens of treatment of kala-azar with sodium stibogluconate in India: A randomised study. *Br Med J (Clin Res Ed)*. 1988;296(6636):1557-1561. doi:10.1136/bmj.296.6636.1557

71. Mueller Y, Nguimfack A, Cavailler P, et al. Safety and effectiveness of amphotericin B deoxycholate for the treatment of visceral leishmaniasis in Uganda. *Ann Trop Med Parasitol*. 2008;102(1):11-19. doi:10.1179/136485908X252142

72. Khalil EAG, Weldegebreal T, Younis BM, et al. Safety and Efficacy of Single Dose versus Multiple Doses of AmBisome® for Treatment of Visceral Leishmaniasis in Eastern Africa: A Randomised Trial. *PLoS Negl Trop Dis*. 2014;8(1):e2613. doi:10.1371/journal.pntd.0002613

73. Davidson RN, di Martino L, Gradoni L, et al. Short-course treatment of visceral leishmaniasis with liposomal amphotericin B (AmBisome). *Clin Infect Dis*. 1996;22(6):938-943. doi:10.1093/clinids/22.6.938

74. Sundar S, Agrawal N, Arora R, Agarwal D, Rai M, Chakravarty J. Short‐Course Paromomycin Treatment of Visceral Leishmaniasis in India: 14‐Day vs 21‐Day Treatment. *Clin Infect Dis*. 2009;49(6):914-918. doi:10.1086/605438

75. Sundar S, Gupta LB, Rastogi V, Agrawal G, Murray HW. Short-course , visceral leishmaniasis treatment with amphotericin emulsion cures. *Trans R Soc Trop Med Hyg*. 2000;94:200-204.

76. Sundar S, Agrawal NK, Sinha PR, Horwith GS, Murray HW. Short-course, low-dose amphotericin B lipid complex therapy for visceral leishmaniasis unresponsive to antimony. *Ann Intern Med*. 1997;127(2):133-137. doi:10.7326/0003-4819-127-2-199707150-00007

77. Sundar S, Jha TK, Thakur CP, Mishra M, Singh VP, Buffels R. Single‐Dose Liposomal Amphotericin B in the Treatment of Visceral Leishmaniasis in India: A Multicenter Study. *Clin Infect Dis*. 2003;37(6):800-804. doi:10.1086/377542

78. Sundar, Shyamgarwal D, Rai M, Murray HW. Single-Dose Liposomal Amphotericin B for Visceral Leishmaniasis in India. *N Engl J Med*. 2010;362(2):504-512.

79. Musa A, Khalil E, Hailu A, et al. Sodium stibogluconate (ssg) & paromomycin combination compared to ssg for visceral leishmaniasis in east africa: A randomised controlled trial. *PLoS Negl Trop Dis*. 2012;6(6). doi:10.1371/journal.pntd.0001674

80. Zijlstra EE, Ali MS, El-Hassan AM, et al. The treatment of kala-azar in the Sudan with sodium stibogluconate: A randomized trial of three dosage regimens. *Trans R Soc Trop Med Hyg*. 1993;87(3):307-309. doi:10.1016/0035-9203(93)90140-L

81. Sundar S, Goyal AK, More DK, Singh MK, Murray HW. Treatment of antimony-unresponsive Indian visceral leishmaniasis with ultra-short courses of amphotericin-B-lipid complex. *Ann Trop Med Parasitol*. 1998;92(7):755-764. doi:10.1080/00034983.1998.11813337

82. Dietze R, Milan E, Berman J, Grogl M, et al. Treatment of Brazilian kala-azar with a short course of amphocil (amphotericin B cholesterol dispersion). *Clin Infect Dis*. 1993;17(6):981-986.

83. Sundar S, Agrawal G, Rai M, et al. Treatment of Indian visceral leishmaniasis with single or daily infusions of low dose liposomal amphotericin B: randomised trial. *BMJ*. 2001;323(7310):419-422. doi:10.1136/bmj.323.7310.419

84. Dietze R, Fagundes S, Brito E, et al. Treatment of Kala-Azar in Brazil With Amphocil(R) (Amphotericin-B Cholesterol Dispersion) for 5 Days. *Trans R Soc Trop Med Hyg*. 1995;89(3):309-311. doi:10.1016/0035-9203(95)90557-X

85. Chunge CN, Owate J, Pamba HO, Donno L. Treatment of visceral leishmaniasis in Kenya by aminosidine alone or combined with sodium stibogluconate. *Trans R Soc Trop Med Hyg*. 1990;84(2):221-225. doi:10.1016/0035-9203(90)90263-E

86. Thakur CP, Kanyok TP, Pandey A, Sinha GP, Messick C, Olliaro P. Treatment of visceral leishmaniasis with injectable paromomycin (aminosidine). An open-label randomized phase-II clinical study. *Trans R Soc Trop Med Hyg*. 2000;94:432-433.

87. Thakur CP, Olliaro P, Gothoskar S, et al. Treatment of visceral leishmaniasis (kala-azar) with aminosidine (=paromomycin)-antimonial combinations, a pilot study in bihar, india. *Trans R Soc Trop Med Hyg*. 1992;86(6):615-616. doi:10.1016/0035-9203(92)90150-B

88. Sundar S, Rosenkaimer F, Makharia MK, et al. Trial of oral miltefosine for visceral leishmaniasis. *Lancet*. 1998;352(9143):1821-1823. doi:10.1016/S0140-6736(98)04367-0

89. Jha TK, Giri YN, Singh TK, Jha S. Use of amphotericin B in drug-resistant cases of visceral leishmaniasis in North Bihar, India. *Am J Trop Med Hyg*. 1995;52(6):536-538. doi:10.4269/ajtmh.1995.52.536

90. Mondal S, Bhattacharya P, Rahaman M, Ali N, Goswami RP. A curative immune profile one week after treatment of Indian Kala-azar patients predicts success with a Short-course liposomal amphotericin B therapy. *PLoS Negl Trop Dis*. 2010;4(7):1-8. doi:10.1371/journal.pntd.0000764

91. Veeken H, Ritmeijer K, Seaman J, Davidson R. A randomized comparison of branded sodium stibogluconate and generic sodium stibogluconate for the treatment of visceral leishmaniasis under field conditions in Sudan. *Trop Med Int Heal*. 2000;5(5):312-317. doi:10.1086/520665

92. Mishra M, Biswas UK, Jha DN, Khan AB. Amphotericin versus pentamidine in antimony-unresponsive kala-azar. *Lancet*. 1992;340(8830):1256-1257. doi:10.1016/0140-6736(92)92952-C

93. Mishra M, Biswas UK, Jha AM, Khan AB. Amphotericin versus sodium stibogluconate in first-line treatment of Indian kala-azar. *Lancet*. 1994;344(8937):1599-1600. doi:10.1016/S0140-6736(94)90406-5

94. Rijal S, Bhandari S, Koirala S, et al. Clinical risk factors for therapeutic failure in kala-azar patients treated with pentavalent antimonials in Nepal. *Trans R Soc Trop Med Hyg*. 2010;104(3):225-229. doi:10.1016/j.trstmh.2009.08.002

95. Thakur CP, Pandey a K, Sinha GP, Roy S, Behbehani K, Olliaro P. Comparison of three treatment regimens with liposomal amphotericin B (AmBisome(R)) for visceral leishmaniasis in India: A randomized dose-finding study. *TransRSoc Trop Med Hyg*. 1996;90(3):319-322. isi:A1996UT79400041

96. Berman JD, Badaro R, Thakur CP, et al. Efficacy and safety of liposomal amphotericin B (AmBisome) for visceral leishmaniasis in endemic developing countries. *Bull World Health Organ*. 1998;76(1):25-32.

97. Maji A, Saha P, Ganguly S, et al. Efficacy of oral miltefosine in visceral leishmaniasis in rural West Bengal, India. *Indian J Pharmacol*. 2012;44(4):500. doi:10.4103/0253-7613.99326

98. Thakur CP, Sinha GP, Sharma V, Pandey AK, Kumar M, Verma BB. Evaluation of amphotericin B as a first line drug in comparison to sodium stibogluconate in the treatment of fresh cases of kala-azar. *Indian J Med Res*. 1993;97(July):170-175.

99. Mishra M, Thakur B, Choudhary M. Metronidazole and indian kalaazar: Results of a clinical trial. *Br Med J (Clin Res Ed)*. 1985;291(6509):1611. doi:10.1136/bmj.291.6509.1611

100. Figueras Nadal M, García de Miguel M, Asensi Botet F, Velasco Bernardo R, Canals Baeza A, Ausín Aoiz I. [Short course treatment for visceral leishmaniasis with liposomal amphotericin B in immunocompetent patients]. *An Pediatr*. 2003;59(6):535-540.

101. Singh UK, Prasad R, Jaiswal BP, Singh PK, Thakur CP. Amphotericin B therapy in children with visceral leishmaniasis: Daily vs. alternate day, a randomized trial. *J Trop Pediatr*. 2010;56(5):321-324. doi:10.1093/tropej/fmp132

102. Castagnola E, Davidson RN, Fiore P, et al. Early efficacy of liposomal amphotericin B in the treatment of visceral leishmaniasis. *Trans R Soc Trop Med Hyg*. 1996;90(3):317-318. doi:10.1016/S0035-9203(96)90270-9

103. Thakur CP, Sinha GP, Sharma V, Pandey AK, Sinha PK, Barat D. Efficacy of amphotericin B in multi-drug resistant kala-azar in children in first decade of life. *Indian J Pediatr*. 1993;60(1):29-36. doi:10.1007/BF02860503

104. Bhattacharya SK, Jha TK, Sundar S, et al. Efficacy and Tolerability of Miltefosine for Childhood Visceral Leishmaniasis in India. *Clin Infect Dis*. 2004;38(2):217-221. doi:10.1086/380638

105. Singh UK, Prasad R, Kumar R, Jaiswal BP. Miltefosine in children with visceral leishmaniasis. *Indian Pediatr*. 2006;43(12):1076-1080.

106. Singh UK, Prasad R, Mishra OP, Jayswal BP. Miltefosine in children with visceral leishmaniasis: A prospective, multicentric, cross-sectional study. *Indian J Pediatr*. 2006;73(12):1077-1080. doi:10.1007/BF02763048

107. Sundar S, Jha TK, Sindermann H, Junge K, Bachmann P, Berman J. Oral miltefosine treatment in children with mild to moderate Indian visceral leishmaniasis. *Pediatr Infect Dis J*. 2003;22(5):434-438. doi:10.1097/01.inf.0000066877.72624.cb

108. Karimi A, Alborzi I, Mahmodi MR, Rakhshani AH, Kadivar MR. Short course anti-leishmania therapy in children with visceral leishmaniasis. *Iran J Med Sci*. 1998;23(1-2):6-10.

109. Di Martino L, Davidson RN, Giacchino R, et al. Treatment of visceral leishmaniasis in children with liposomal amphotericin B. *J Pediatr*. 1997;131(2):271-277.

110. Syriopoulou V, Daikos GL, Theodoridou M, et al. Two doses of a lipid formulation of amphotericin B for the treatment of Mediterranean visceral leishmaniasis. *Clin Infect Dis*. 2003;36(5):560-566. doi:10.1086/367843

111. Haidar NA, Diab A-BL, EL-Sheikh AM. Visceral Leishmaniasis in children in the Yemen. *Saudi Med J*. 2001;22(6):516-519.

112. Tobaigy M, Mejer J, Peitersen B, Ansari M, Poll P. A comparative study of allopurinol and pentostam in the treatment of visceral leishmaniasis. *Adv Exp Med Biol*. 1986;195 Pt A(Table 3):471-473.

113. Sahay AS, Jha T. Amphotericin B in Resistant Visceral Leishmaniasis. *Indian Pediatr*. 1996;33(June):1995-1997.

114. Mishra M, Singh MP, Choudhury D, Singh VP, Khan AB. Amphotericin B for second-line treatment of Indian kala-azar. *Lancet*. 1991;337(8746):926. doi:10.1016/0140-6736(91)90268-T

115. Das VNR, Ranjan A, Bimal S, et al. Magnitude of unresponsiveness to sodium stibogluconate in the treatment of visceral leishmaniasis in Bihar. *Natl Med J India*. 2005;18(3):131-133.

116. Chowdhury S, Haque F, Al-Masum A, El Harith A, Karim E. Positive response to sodium antimony gluconate administration in visceral leishmaniasis seropositive patients. *Am J Trop Med Hyg*. 1991;44(4):390-393. doi:10.4269/ajtmh.1991.44.390

117. Rees PH, Kager PA, Wellde BT, Hockmeyer WT. The response of Kenyan kala-azar to treatment with sodium stibogluconate. *Am J Trop Med Hyg*. 1984;33(3):357-361. doi:10.4269/ajtmh.1984.33.357

118. Rijal S, Chappuis F, Singh R, et al. Treatment of visceral leishmaniasis in south-eastern Nepal: Decreasing efficacy of sodium stibogluconate and need for a policy to limit further decline. *Trans R Soc Trop Med Hyg*. 2003;97(3):350-354. doi:10.1016/S0035-9203(03)90167-2

119. Thakur CP, Kumar P, Kumar N, Singh GN, Singh AK, Narain S. A randomized comparison of classical mode of administration of amphotericin B with its newer modes of administration in kala-azar. *J Assoc Physicians India*. 1998;46(9):779-783.

120. Das VNR, Ranjan A, Sinha AN, et al. A Randomized Clinical Trial of Low Dosage Combination of Pentamidine and Allopurinol in the Treatment of Antimony Unresponsive Cases of Visceral Leishmaniasis. *J Assoc Physicians India*. 2001;49(JUNE):609-613.

121. Sundar S, Goyal AK, Mandal AK, Makharia MK, Singh VP, Murray HW. Amphotericin B Lipid Complex in the Management of Antimony Unresponsive Indian Visceral Leishmaniasis. *J Assoc Physicians India*. 1999;47(3):186-188.

122. Jha SN, Singh NK, Jha TK. Changing response to diamidine compounds in cases of kala-azar unresponsive to antimonial. *J Assoc Physicians India*. 1991;39(4):314-316.

123. Singh NK, Jha TK, Singh IJ, Jha S. Combination therapy in Kala-azar. *J Assoc Physicians India*. 1995;43(5):319-320.

124. Giri OP, Singh AN. Experience with amphotericin B in sodium stibogluconate -unresponsive cases of visceral Leishmaniasis in north Bihar. *J Assoc Physicians India*. 1994;42(9):690-691.

125. Lal SK, Lal R, Lal S, Lal R, Lal BN. Experience with roxithromycin in visceral leishmaniasis in North Bihar. *J Assoc Physicians India*. 1996;44(9):615-617.

126. Giri OP. Treatment of visceral Leishmaniasis unresponsive to pentamidine with amphotericin B. *J Assoc Physicians India*. 1994;42(9):688-689.

127. Thakur CP. Epidemiological, clinical and therapeutic features of Bihar kala-azar (including post kala-azar dermal leishmaniasis). *Trans R Soc Trop Med Hyg*. 1984;78(3):391-398. doi:10.1016/0035-9203(84)90131-7

128. Jha BB. Fluconazole in visceral leishmaniasis. *Indian Pediatr*. 1998;35:268-269.

129. Ostyn B, Hasker E, Dorlo TPC, et al. Failure of miltefosine treatment for visceral leishmaniasis in children and men in South-East Asia. *PLoS One*. 2014;9(6). doi:10.1371/journal.pone.0100220

130. Sinha PK, Roddy P, Palma PP, et al. Effectiveness and safety of liposomal amphotericin b for visceral leishmaniasis under routine program conditions in Bihar, India. *Am J Trop Med Hyg*. 2010;83(2):357-364. doi:10.4269/ajtmh.2010.10-0156

131. Mueller M, Ritmeijer K, Balasegaram M, Koummuki Y, Santana MR, Davidson R. Unresponsiveness to AmBisome in some Sudanese patients with kala-azar. *Trans R Soc Trop Med Hyg*. 2007;101(1):19-24. doi:10.1016/j.trstmh.2006.02.005

132. Cota GF, de Sousa MR, de Mendonça ALP, et al. Leishmania-HIV Co-infection: Clinical Presentation and Outcomes in an Urban Area in Brazil. *PLoS Negl Trop Dis*. 2014;8(4):2-8. doi:10.1371/journal.pntd.0002816

133. Sudarshan M, Weirather JL, Wilson ME, Sundar S. Study of parasite kinetics with antileishmanial drugs using real-time quantitative PCR in Indian visceral leishmaniasis. *J Antimicrob Chemother*. 2011;66(8):1751-1755. doi:10.1093/jac/dkr185

134. Sundar S, Kumar P, Makharia M, et al. Atovaquone alone or with fluconazole as oral therapy for Indian kala-azar. *Clin Infect Dis*. 1998;27(1):215-216. doi:10.1086/517679

135. Adam GK, Abdulla MA, Ahmed AA, Adam I. Maternal and perinatal outcomes of visceral leishmaniasis (kala-azar) treated with sodium stibogluconate in eastern Sudan. *Int J Gynecol Obstet*. 2009;107(3):208-210. doi:10.1016/j.ijgo.2009.08.002

136. Shahian M, Alborzi A. Effect of meglumine antimoniate on the pancreas during treatment of visceral leishmaniasis in children. *Med Sci Monit*. 2009;15(6):CR290-3. doi:869677 [pii]

137. Villanueva JL, Alarcon A, Bernabeu-Wittel M, et al. Prospective evaluation and follow-up of European patients with visceral leishmaniasis and HIV-1 coinfection in the era of highly active antiretroviral therapy. *Eur J Clin Microbiol Infect Dis*. 2000;19(10):798-801.

138. Thakur CP, Kumar M. Observations on the effect of verapamil with sodium stibogluconate in kala-azar. *Trop Geogr Med*. 1992;44(1-2):15-18.

139. Thakur CP. Sodium antimony gluconate, amphotericin, and myocardial damage. *Lancet*. 1998;351(9120):1929-1930. doi:10.1016/S0140-6736(05)78613-X

140. Goswami RP, Goswami RP, Das S, Satpati A, Rahman M. Short-course treatment regimen of indian visceral leishmaniasis with an indian liposomal amphotericin b preparation (fungisome^TM^). *Am J Trop Med Hyg*. 2016;94(1):93-98. doi:10.4269/ajtmh.14-0657

141. Jamil KM, Haque R, Rahman R, et al. Effectiveness Study of Paromomycin IM Injection (PMIM) for the Treatment of Visceral Leishmaniasis (VL) in Bangladesh. *PLoS Negl Trop Dis*. 2015;9(10):1-11. doi:10.1371/journal.pntd.0004118

142. Sundar S, Singh A, Rai M, Chakravarty J. Single-dose indigenous liposomal amphotericin B in the treatment of Indian visceral leishmaniasis: A phase 2 study. *Am J Trop Med Hyg*. 2015;92(3):513-517. doi:10.4269/ajtmh.14-0259

143. Sundar S, Pandey K, Thakur CP, et al. Efficacy and Safety of Amphotericin B Emulsion versus Liposomal Formulation in Indian Patients with Visceral Leishmaniasis: A Randomized, Open-Label Study. *PLoS Negl Trop Dis*. 2014;8(9):1-7. doi:10.1371/journal.pntd.0003169

144. Mondal D, Alvar J, Hasnain MG, et al. Efficacy and safety of single-dose liposomal amphotericin B for visceral leishmaniasis in a rural public hospital in Bangladesh: A feasibility study. *Lancet Glob Heal*. 2014;2(1):e51-e57. doi:10.1016/S2214-109X(13)70118-9

145. Rashid JR, Wasunna KM, Gachihi GS, Nyakundi PM, Mbugua J, Kirigi G. The efficacy and safety of ketoconazole in visceral leishmaniasis. *East Afr Med J*. 1994;71(6):392-395.

146. Sundar S, Singh A, Agrawal N, Chakravarty J. Effectiveness of Single-Dose Liposomal Amphotericin B in Visceral Leishmaniasis in Bihar. *Am J Trop Med Hyg*. 2019;101(4):795-798. doi:10.4269/ajtmh.19-0179

147. Diro E, Blesson S, Edwards T, et al. A randomized trial of AmBisome monotherapy and AmBisome and miltefosine combination to treat visceral leishmaniasis in HIV co-infected patients in Ethiopia. *PLoS Negl Trop Dis*. 2019;13(1):1-19. doi:10.1371/journal.pntd.0006988

148. Mbui J, Olobo J, Omollo R, et al. Pharmacokinetics, safety and efficacy of an allometric miltefosine regimen for the treatment of visceral leishmaniasis in Eastern African children: an open-label, phase-II clinical trial. *Clin Infect Dis*. Published online 2018. doi:10.1093/cid/ciy747

149. Borges MM, da Silva Pranchevicius MC, Noronha EF, Romero GAS, Carranza-Tamayo CO. Efficacy and safety of amphotericin B deoxycholate versus N-methylglucamine antimoniate in pediatric visceral leishmaniasis: An open-label, randomized, and controlled pilot trial in Brazil. *Rev Soc Bras Med Trop*. 2017;50(1):67-74. doi:10.1590/0037-8682-0455-2016

150. Wasunna M, Njenga S, Balasegaram M, et al. Efficacy and Safety of AmBisome in Combination with Sodium Stibogluconate or Miltefosine and Miltefosine Monotherapy for African Visceral Leishmaniasis: Phase II Randomized Trial. *PLoS Negl Trop Dis*. 2016;10(9):1-18. doi:10.1371/journal.pntd.0004880

151. Rahman R, Goyal V, Haque R, et al. Safety and efficacy of short course combination regimens with AmBisome, miltefosine and paromomycin for the treatment of visceral leishmaniasis (VL) in Bangladesh. *PLoS Negl Trop Dis*. 2017;11(5):1-15. doi:10.1371/journal.pntd.0005635

152. Romero GAS, Costa DL, Costa CHN, et al. Efficacy and safety of available treatments for visceral leishmaniasis in Brazil: A multicenter, randomized, open label trial. *PLoS Negl Trop Dis*. 2017;11(6):1-25. doi:10.1371/journal.pntd.0005706

153. Goswami RP, Rahman M, Das S, Tripathi SK, Goswami RP. Combination therapy against Indian visceral leishmaniasis with liposomal amphotericin B (FungisomeTM) and short-course miltefosine in comparison to miltefosine monotherapy. *Am J Trop Med Hyg*. 2020;103(1):308-314. doi:10.4269/ajtmh.19-0931

154. Alborzi A, Pouladfar G, Attar A, et al. Effectiveness of short-course meglumine antimoniate (Glucantime®) for treatment of visceral leishmaniasis: A 13-year, multistage, non-inferiority study in Iran. *Am J Trop Med Hyg*. 2017;96(1):182-189. doi:10.4269/ajtmh.16-0345

155. Pandey K, Pal B, Siddiqui NA, et al. Efficacy and Safety of Liposomal Amphotericin B for Visceral Leishmaniasis in Children and Adolescents at a Tertiary Care Center in Bihar, India. *Am J Trop Med Hyg*. 2017;97(5):1498-1502. doi:10.4269/ajtmh.17-0094

156. Pandey K, Ravidas V, Siddiqui NA, et al. Pharmacovigilance of miltefosine in treatment of visceral leishmaniasis in endemic areas of Bihar, India. *Am J Trop Med Hyg*. 2016;95(5):1100-1105. doi:10.4269/ajtmh.16-0242

157. Kimutai R, Musa AM, Njoroge S, et al. Safety and Effectiveness of Sodium Stibogluconate and Paromomycin Combination for the Treatment of Visceral Leishmaniasis in Eastern Africa: Results from a Pharmacovigilance Programme. *Clin Drug Investig*. 2017;37(3):259-272. doi:10.1007/s40261-016-0481-0

158. Goyal V, Mahajan R, Pandey K, et al. Field safety and effectiveness of new visceral leishmaniasis treatment regimens within public health facilities in Bihar, India. *PLoS Negl Trop Dis*. 2018;12(10):e0006830. doi:10.1371/journal.pntd.0006830

159. Sinha KK, Basant A, Patel A. A study on efficacy and tolerability of miltefosine for childhood visceral leishmaniasis in a tertiary care centre in eastern bihar. *Indian J Appl Res*. 2019;9(2):33-36.

160. Ekram MR, Amin MR, Hasan MJ, et al. Efficacy and safety of single-dose liposomal amphotericin B in patients with visceral leishmaniasis in Bangladesh: a real-life experience. *J Parasit Dis*. 2021;45(4):903-911. doi:10.1007/s12639-021-01379-w
